# HIV-1 Populations in Semen Arise through Multiple Mechanisms

**DOI:** 10.1371/journal.ppat.1001053

**Published:** 2010-08-19

**Authors:** Jeffrey A. Anderson, Li-Hua Ping, Oliver Dibben, Cassandra B. Jabara, Leslie Arney, Laura Kincer, Yuyang Tang, Marcia Hobbs, Irving Hoffman, Peter Kazembe, Corbin D. Jones, Persephone Borrow, Susan Fiscus, Myron S. Cohen, Ronald Swanstrom, and the Center for HIV/AIDS Vaccine Immunology

**Affiliations:** 1 UNC Center for AIDS Research, University of North Carolina, Chapel Hill, North Carolina, United States of America; 2 Department of Medicine, University of North Carolina, Chapel Hill, North Carolina, United States of America; 3 Lineberger Cancer Center, University of North Carolina, Chapel Hill, North Carolina, United States of America; 4 The Edward Jenner Institute for Vaccine Research, Compton, Berkshire, United Kingdom; 5 Department of Biology, University of North Carolina, Chapel Hill, North Carolina, United States of America; 6 Department of Microbiology and Immunology, University of North Carolina, Chapel Hill, North Carolina, United States of America; 7 Baylor Pediatric Center of Excellence, Lilongwe, Malawi; 8 Department of Biochemistry and Biophysics, University of North Carolina, Chapel Hill, North Carolina, United States of America; NIH/NIAID, United States of America

## Abstract

HIV-1 is present in anatomical compartments and bodily fluids. Most transmissions occur through sexual acts, making virus in semen the proximal source in male donors. We find three distinct relationships in comparing viral RNA populations between blood and semen in men with chronic HIV-1 infection, and we propose that the viral populations in semen arise by multiple mechanisms including: direct import of virus, oligoclonal amplification within the seminal tract, or compartmentalization. In addition, we find significant enrichment of six out of nineteen cytokines and chemokines in semen of both HIV-infected and uninfected men, and another seven further enriched in infected individuals. The enrichment of cytokines involved in innate immunity in the seminal tract, complemented with chemokines in infected men, creates an environment conducive to T cell activation and viral replication. These studies define different relationships between virus in blood and semen that can significantly alter the composition of the viral population at the source that is most proximal to the transmitted virus.

## Author Summary

The work described in this report is directed at how HIV-1 viral RNA populations differ between the blood plasma and male genital tract in established infection. This site is of special interest since it is the proximal source of most transmissions of HIV-1. Thus, lessons learned about HIV-1 in the seminal tract are directly relevant to the mechanism of HIV-1 transmission. We have used single genome amplification to generate viral sequences from paired blood and semen samples in men with chronic HIV-1 infection. When compared to viral populations in blood plasma, we observe that virus in the seminal plasma can be equilibrated, clonally-amplified, or compartmentalized. We have also performed a characterization of the cytokine and chemokine milieu in these two compartments. We report a dramatic concentration of immune modulators in the seminal plasma relative to the blood, and these likely enhance the potential for viral replication in this compartment by creating an environment where target cells are kept in an activated state. These data define new and distinct features of virus:host interactions and represent a significant advance in our understanding of HIV-1 replication in the male genital tract.

## Introduction

Sexual transmission of the human immunodeficiency virus type 1 (HIV-1) is the most common mode of transmission worldwide. During sexual transmission, genital secretions are the most proximal source of the transmitted virus. Thus, an understanding of the virus at these sites is central to understanding the transmission event and the nature of the transmitted virus. In this study we have explored the nature of viral populations in seminal plasma.

Virus enters the male genital tract during primary infection [1]–[5]. Initially, the virus found in the semen is similar, if not identical, to that found in the blood [6], [7]. During primary infection the viral RNA load is elevated in both the blood and the semen [1], [3]. The probability of transmission is related to the level of virus in the blood of the donor [8]–[11] and, based on a small cohort, to the level of virus in the semen [12]. Factors that induce inflammation in the seminal tract, such as sexually transmitted infections (STI), can raise the level of virus in semen [13], and this may contribute to the transmission of HIV-1 by the sexual route [14]. In addition, the endogenous semen-derived enhancer of virus infection (SEVI), a fragment of prostatic acid phosphatase, has been shown to increase infectious viral titers in vitro by several orders of magnitude [15].

The presence of virus in semen raises the possibility that virus found in semen could be the product of replication within the seminal tract. CD4+ T cells are found in semen indicating the presence of target cells that could support replication [16], [17]. SIV-infected macaques have infected cells within the tissues of the seminal tract [18], [19], supporting the possibility for local viral replication. Several studies have examined the relationship between viral populations found in blood and semen and noted differences (i.e. compartmentalization) using discordant drug resistance markers [20]–[24], differences in population markers [25], [26], or phylogenetic analysis [27]–[32].

In this study we have carried out a detailed examination of the viral populations in semen, comparing the *env* gene in blood plasma and seminal plasma. The men were therapy-naive and chronically infected with subtype C (n = 12) or subtype B (n = 4) HIV-1. We found a varied and complex relationship between these two compartments which suggests multiple types of biological phenomena. There is evidence for the direct import of virus from the blood to the semen, evidence for clonal amplification of a subset of genotypes within the seminal tract, and evidence for sustained replication and distinct evolution of virus within the seminal tract resulting in compartmentalization. The latter two of these phenomena result in seminal plasma viral populations that are distinct from those found in the blood, and thus distinct at the site proximal to transmission. Furthermore, semen is enriched in cytokines which may increase the potential for independent viral replication within the seminal tract.

## Results

### Cohort characteristics and generation of sequences

In this study, we examined viral populations and cytokine/chemokine relationships in paired blood and semen samples collected from men with chronic HIV-1 infection in Lilongwe, Malawi (n = 12) [33], or from the CHAVI 001 clinical study (n = 4). We utilized a cohort of men without urethritis to minimize any potential confounders on viral loads, viral populations, or cytokine profiles. The clinical parameters of their HIV-1 infection are shown in Table 1. There was no evidence of urethritis in the dermatology clinic subjects, although the diagnosed cases of syphilis (1) and trichomonas (4) were treated with appropriate antibiotics (Table 1). In addition, blood and semen samples were obtained from twelve HIV-1-negative men without STIs from North Carolina and from 6 men from Malawi to serve as a control for the cytokine and chemokines analyses.

**Table 1:** Patient cohort to analyze blood and semen viral populations in established infection.

ID^a^	Subtype	Blood VL^b^	Semen VL^b^	CD4 Count^c^	Blood seq	Semen seq	Semen Amplification^d^	Semen Compartment
C007	C	4.9	3.6	190	29	23	Amp	Equilibrated
C009	C	5.1	5.3	505	26	28	Amp	Equilibrated
C011	C	5.4	4.4	67	30	28	No Amp	Equilibrated
C012	C	5.4	6.7	863	26	20	Amp	Equilibrated
C018	C	6.2	6.4	116	32	30	No Amp	Compartmentalized
C019	C	5.7	5.8	599	31	44	Amp	Equilibrated
C047	C	5.1	5.3	291	19	27	Amp	Compartmentalized
C070	C	5.4	5.1	172	34	30	Amp	Equilibrated
C083	C	4.8	5.4	423	18	26	No Amp	Compartmentalized
C109	C	4.8	5.7	328	18	25	Amp	Equilibrated
C111	C	5.0	5.9	210	17	27	No Amp	Equilibrated
C113	C	4.6	4.6	279	29	28	Amp	Compartmentalized
700010333	B	4.2	4.7	501	23	33	Amp	Equilibrated
700010501	B	4.3	4.5	514	18	14	Amp	Equilibrated
701010380	B	5.1	3.6	129	18	15	Amp	Compartmentalized
700011145	B	4.7	4.2	356	15	20	Amp	Equilibrated

a C011 had syphilis without urethritis; C070, C083, C109, and C113 had asymptomatic trichomonas infection without urethritis.

b Log_10_ of HIV-1 viral RNA load (copies/ml).

c Number of CD4^+^ T lymphocytes/mm^3^.

d Presence or absence of clonal outgrowth or amplification in seminal plasma.

We used viral RNA extracted from blood plasma and seminal plasma to generate cDNAs to use as a template in the single genome amplification (SGA) protocol of the viral *env* gene [34]–[36]. The use of viral RNA allows for the examination of contemporaneously replicating virus. A mean of 27 amplicons were analyzed per sample, with a range of 15 to 34 in the blood and 14 to 44 amplicons in the seminal plasma. Sampling this number of genomes provides a 95% chance to detect a subpopulation in the range of 10–15%, and provides reasonable power to estimate the relative proportions of the major variants in the population [35]. The sequence of the entire *env* gene was determined for each amplicon. The use of SGA precludes PCR recombination as a source of confusion about the relationship between the viral genomes present in each sample [36].

### Viral populations can be well equilibrated between blood and semen

**Figure 1:**
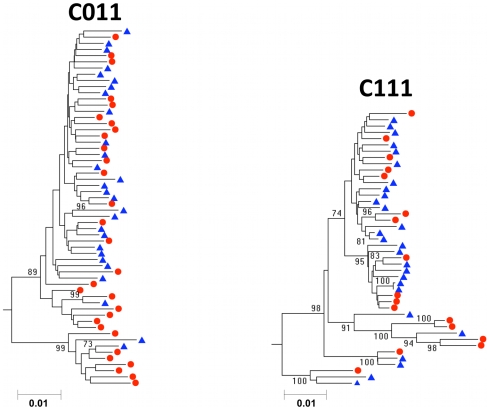
Neighbor-joining tree of SGA-derived *env* amplicons for two patients with HIV-1 subtype C demonstrating equilibration between blood and semen. Blood SGA-*env* sequences (red circles) and semen SGA-*env* sequences (blue triangles). Bootstrap values ≥70 are shown. Genetic distance is indicated at the bottom of the figure, and represents the number of nucleotide substitutions per site. An outgroup was included to root the tree but is not shown.

Figure 1 depicts the phylogenetic analysis of viral sequences in the blood and semen for subjects C011 and C111. There was a diverse population in the blood and this diversity was fully represented in the semen. Furthermore, the complexity of the sequences in the blood, where no two sequences were identical, was also represented in the semen. We conclude that in these subjects there was no compartmentalization in the seminal tract. If there were local replication of virus in the seminal tract of these subjects it must have represented the full complexity of the virus in the blood. Alternatively, this virus did not replicate in the seminal tract but rather was imported from the blood. In either scenario, the viral populations in the blood and semen were essentially identical, representing well-equilibrated populations.

### Detection of clonal amplification in the seminal compartment

**Figure 2:**
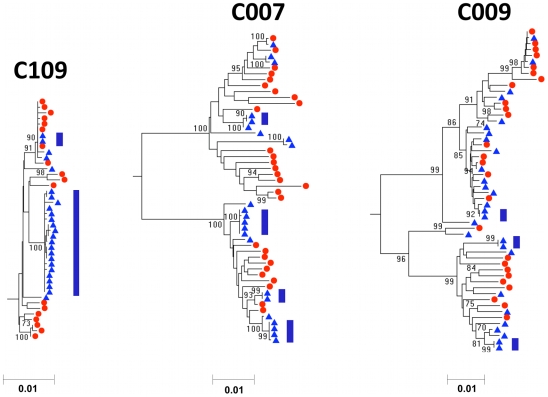
Neighbor-joining tree of SGA-derived *env* amplicons for three patients with HIV-1 subtype C demonstrating clonal amplification of identical or nearly identical sequences within semen. Blood SGA-*env* sequences (red circles) and semen SGA-*env* sequences (blue triangles). Vertical blue bars highlight clonal amplification of specific variants. Bootstrap values ≥70 are shown. An outgroup was included to root the tree but is not shown.

A different phylogenetic pattern was detected in subjects C007, C009, C012, C019, C070, and C109 (Fig. 2 and Fig. S1). Similar to the subjects where blood and semen populations were well-equilibrated, these subjects had viral populations in the semen that represented the full diversity of the virus in the blood. In addition, the blood populations were highly complex and consistent with a diverse viral population, with no sampling of identical blood sequences with the exception of patient C109. However, there was an additional feature of the viral populations in the semen of these subjects that distinguished them from the virus in the blood. In these subjects sampling of the viral population in semen resulted in examples where identical or nearly identical sequences were observed (Fig. 2, Fig. S1, Fig. S3, and Fig. S4-S15). Patient C109 had a clade of identical/nearly identical sequences that comprised nearly 75% of the entire semen viral population. Similarly, patient C009 had three duplicated viral variants that each comprised <10% of the semen population, indicating a broad range in the amount of sequence duplication that can exist within semen. We term this phenomenon clonal amplification, and because of the nature of the SGA strategy, this cannot be the result of PCR resampling since each amplicon was generated from a single template.

### Autonomous replication in the seminal compartment results in compartmentalization

**Figure 3:**
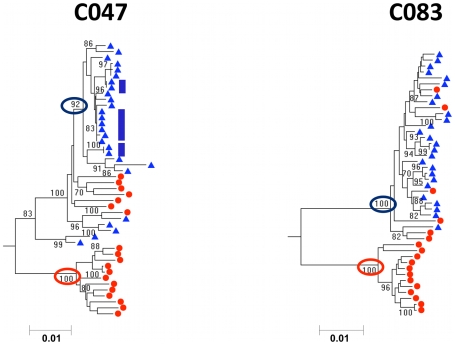
Neighbor-joining tree of SGA-derived *env* amplicons for two patients with HIV-1 subtype C demonstrating compartmentalization between blood and semen. Blood SGA-*env* sequences (red circles) and semen SGA-*env* sequences (blue triangles). Ovals highlight clades of compartmentalized sequences in the semen or blood. Vertical blue bars highlight clonal amplification of specific variants. Bootstrap values ≥70 are shown. An outgroup was included to root the tree but is not shown.

In four of the 12 subjects with subtype C HIV-1 (C047, C083, C018, and C113), a third relationship between the viral populations in the blood and semen was seen. For these subjects there was a deep branch point with high bootstrap support in the phylogenetic tree separating sequences found in the blood from sequences found in the semen (Fig. 3 and Supplemental Fig. S2). In addition to visual inspection of the phylogenetic trees to identify semen clades with long branch lengths with high bootstrap support, the presence of compartmentalized sequences was confirmed with the Slatkin-Maddison statistical [37] and correlation coefficient tests [38] available through Hypothesis testing through Phylogenies (HyPhy) [39]. Previous analyses have revealed that there is no gold standard from the variety of statistical measures available for detecting compartmentalization; therefore, multiple tests are recommended to determine the existence of compartmentalization [40]. Compartmentalization tests were performed with all viral sequences, and after removal of duplicated sequences since amplified variants in the semen can increase both the frequency of compartmentalization calls and the statistical support for those calls (Supplemental Table S2). Thus, compartmentalization of these viral populations was observed in subjects C047, C083, C018, and C113 and indicates an autonomously replicating subpopulation in the seminal tract that followed a distinct evolutionary pathway. As a result of this compartmentalized subpopulation, the virus in the semen was genetically distinct from the virus in the blood. Two subjects (C083 and C018) had compartmentalization of semen-derived sequences without clonal amplification (Fig. 3 and Fig. S2). In addition, two subjects (C047 and C113) had both clonal amplification and compartmentalization of semen-derived sequences (Fig. 3, Fig. S2, Fig. S8, and Fig. S11). Similar to the previous subjects with semen clonal amplification (with the exception of C109 as previously mentioned), there were no duplicated blood sequences. Thus, these data indicate that the male genital tract is capable of supporting complex viral populations, and that compartmentalization and amplification can occur independently.

### Equilibration, amplification, and compartmentalization are characteristics of HIV-1 blood and semen populations in different clades

**Figure 4:**
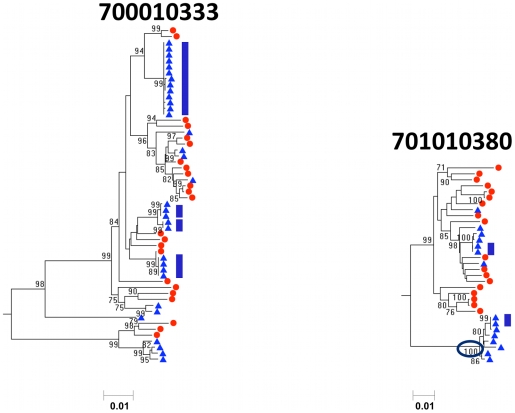
Neighbor-joining tree of SGA-derived *env* amplicons for two patients with HIV-1 subtype B demonstrating amplification and equilibration in the left panel, and amplification and compartmentalization of seminal sequences in the right panel. Blood SGA-*env* sequences (red circles) and semen SGA-*env* sequences (blue triangles). The oval highlights clades of compartmentalized sequences in the semen. Vertical blue bars highlight clonal amplification of specific variants. Bootstrap values ≥70 are shown. An outgroup was included to root the tree but is not shown.

In addition to the 12 men with HIV-1 subtype C infection, we analyzed blood and semen plasma viral RNA populations from 4 men with subtype B infection (Fig. 4, Fig. S3, Fig. S12-15). Each of the four had identical sequences (clonal amplification) in the seminal plasma that ranged from <10% to one-third of the semen viral population. In contrast, none of the patients had identical sequences in the blood plasma. In addition, three of the men had equilibrated blood and seminal plasma sequences: 700010333, 700010501, and 700011145; whereas, one of the 4 subtype B infected men (700010380) had significant compartmentalization of semen-derived sequences. Thus, clonal amplification and compartmentalization within the seminal plasma is a common feature of HIV-1 of different subtypes.

### Evolutionary history of viral *env* gene sequences in semen

We carried out an analysis for each subject using Bayesian Evolutionary Analysis by Sampling Trees (BEAST) [41] to estimate the time to most recent common ancestor (TMRCA) of the amplified variants, and/or the TMRCA of compartmentalized variants using maximum likelihood trees. Of note, the topologies of the neighbor-joining and maximum likelihood trees were very similar (data not shown), indicating that these two different phylogenetic methods produced concordant results in their evolutionary models.

As a control to compare the BEAST estimates to known values obtained from previously published sequence data sets, a separate analysis was performed using a subset of published longitudinal *env* sequences (Fig. S16) [42]. From this data set, we calculated the time of divergence using C2-V5 *env* sequences obtained from longitudinal plasma samples at 3, 29, 42, 58, 70, and 100 months post-seroconversion; BEAST estimates of 10, 34, 49, 147, 144, and 204 months, respectively, were observed with a high coefficient of determination (R^2^ = 0.9155). Thus, in the setting of chronic HIV-1 infection, the observed BEAST estimates were similar to the expected values for periods up to several years, but there is a trend to overestimate time periods greater than four years by approximately two-fold.

Next, we determined the TMRCA of amplified and/or compartmentalized variants within seminal plasma. The TMRCA for the oligoclonal amplifications within the seminal compartment ranged from 1 to 375 days, with a mean of 57 days, indicating recent divergence. In contrast to the short evolutionary times observed with the semen variants displaying oligoclonal amplification, the subjects with significant semen compartmentalization had divergence estimates from 1.5 to 9.7 years, with a mean of 5.2 years. If clonally-amplified sequences were used only once, there was negligible effect on the TMRCA of the entire tree, or the TMRCA of amplified or compartmentalized variants (data not shown). Thus, the TMRCA of the clonally amplified variants tends to be relatively short in contrast to compartmentalized variants, which represent more distant divergence. However, we do not know if the rate of evolution in the semen is comparable to the blood adding additional uncertainty to the accuracy of the absolute values generated with the BEAST analysis.

To determine if populations were evolving randomly under neutral evolution, a Tajima's neutrality test was performed using DnaSP [43]. Fifteen of the 16 patients showed no evidence of selection (P values >0.10); however, C019 had a Tajima's D of -2.1 (P value <0.05) implying either population size expansion, or positive selection. Thus, a coalescent model of viral evolution as assumed by BEAST remains valid for the majority of patients. In the case of C019, the violation of a coalescent model was most likely due to the blood compartment (Tajima's D of -1.82, P value <0.05) vs. the semen compartment (Tajima's D of -1.60, P value >0.05). Taken together, these data suggest that BEAST is a robust tool to compare the TMRCA of amplified and compartmentalized variants for the majority of the patients that were analyzed.

### Enrichment of cytokines and chemokines in semen

**Figure 5:**
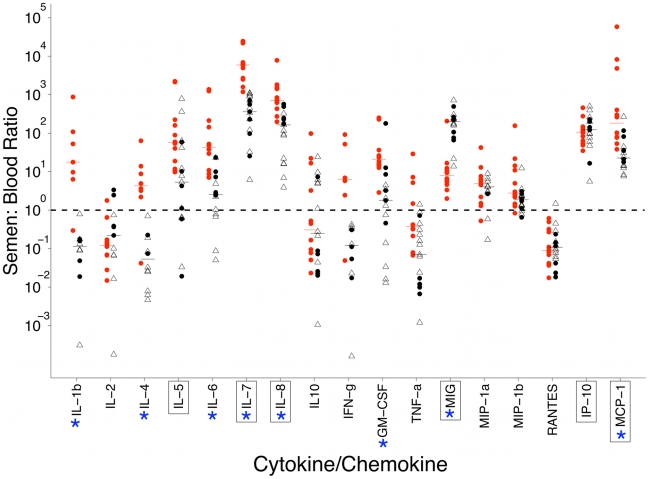
Scatter plot of semen:blood analyte ratios of 17 cytokines and chemokines in HIV-1 infected (red circles) (n = 12), uninfected Malawi men (black circles) (n = 6), and uninfected US men (black triangles) (n = 12). Values that were below the lower limit of detection were reported as the mid-point between the lower level of detection and zero. Semen:blood ratios were excluded if both compartments were below the level of detection; thus, IL12 and IL13 were not included in the analysis. In the infected men, there were 6 to 11 subjects with semen:blood analyte ratios for IL-1b, IL-2, IL-4, and IFN-g, whereas the remaining cytokines and chemokines had data from all 12 infected men. In addition, after exclusion of values below the limit of detection in both semen and blood compartments, we quantified the semen:blood ratio for each cytokine and chemokine from a range of 10 to 17 HIV-1 uninfected men. The dashed line illustrates an equivalent semen:blood analyte ratio. Horizontal lines represent median values. Blue asterisks denote significant differences between infected and uninfected men, P value from Mann-Whitney test <0.003 (to correct for multiple comparisons). Boxed cytokines/chemokines have median semen:blood analyte ratios >5 in both HIV-1 infected and uninfected men.

To determine if the seminal plasma has a distinct immunologic profile relative to blood plasma, we measured the levels of nineteen cytokines and chemokines in the paired blood and semen samples from 12 of the men with chronic HIV-1 subtype C infection. As a control, we measured cytokines and chemokines from paired blood and semen samples in 12 uninfected men from the US and 6 uninfected men from Malawi without STIs. There were two features of the patterns of cytokines and chemokines (Fig. 5) that are noteworthy. First, a subset of cytokines and chemokines (IL-5, IL-7, IL-8, MIG, IP-10, and MCP-1) were concentrated in the semen of uninfected men with median levels that were 5 to approximately 1000 fold greater than in the blood; none of the remaining cytokines or chemokines was as high as five-fold concentrated in the semen (Fig. 5). Second, for seven of the cytokines and chemokines (IL-1b, IL-4, IL6, IL-7, IL-8, GM-CSF, and MCP-1) there was a significant increase in the semen:blood ratio of HIV-infected subjects compared to the uninfected subjects; conversely, MIG was significantly decreased in the infected subjects. Although our small sample size prevented a robust analysis, there were no cytokine correlates with amplification or compartmentalization of HIV-1 sequences in the semen. Moreover, there were no correlates with cytokine levels, and HIV-1 viral loads, amplification, compartmentalization, or the presence of asymptomatic STIs that were detected in five of the HIV-infected men (data not shown, although the small sample size and the intersubject variability precludes an assessment beyond more general trends).

## Discussion

The seminal compartment is the source of the transmitted virus in a majority of the transmission events for HIV-1. Thus, an understanding of the biology of HIV-1 in the seminal tract is integral to understanding the biology of transmission, and a comparison of blood and seminal sequences is critical to increase our knowledge of viral dynamics. We have used viral sequence populations to examine the dynamic relationship between virus and host in the seminal tract, and identify multiple mechanisms by which HIV-1 populations exist in the male genital tract. A significant limitation of this study is that it is cross-sectional, involving a single time point. Another limitation of the current work is that there are no proviral sequences from semen cells to define the source of the amplified or compartmentalized variants.

Previous work has identified paired blood and semen samples where the viral populations were discordant. In some cases this involved a comparison of viral RNA in blood plasma and seminal plasma, or a comparison of the sequences in viral DNA in blood cells and seminal cells [21]–[26], [28], [29], [32]. While these studies clearly established the potential for the virus to become compartmentalized, in most cases there were two potential limitations intrinsic to the experimental approach: the possibility of recombination of viral sequences during PCR which would introduce artifacts into the phylogenetic analysis, and the analysis of a fairly small number of viral genomes in each population precluding a comparison of the population structure. As a result the phenomenon of compartmentalization has been described as a dichotomous state, i.e. the presence or absence of compartmentalized viral populations. However, in subjects where there is equilibration in the seminal tract over the entire range of complexity in the blood compartment, virus in the semen is most easily explained by the direct import of virus into the seminal plasma from blood, perhaps with no local replication of this population.

The enrichment of cytokines and chemokines in the seminal tract (Fig. 5) likely contributes to an environment that is supportive of HIV-1 replication. Our data, as well as others [44], [45], show that in the absence of HIV-1 infection several cytokines and chemokines are enriched, suggesting that the seminal tract maintains a constitutive state of innate immune activation. This state is exacerbated with HIV-1 infection where the concentration of a broader array of cytokines and chemokines indicates both innate and adaptive responses shaping the environment [46]. Thus, target CD4+ T cells and macrophages are likely to be in an activated state in this environment, enhancing their ability to support viral replication.

In several subjects (C109 and 701010380) there is some evidence for clonal amplification of sequences in the blood, with this being more pronounced in C109. However, we do not know if the mechanism causing selective outgrowth in the blood is the same as that in the seminal tract, and in these subjects it is rare in the blood compared to the detection of clonal amplification in the semen in 12 of 16 men. The detection of clonal amplification within the seminal compartment raises several important questions. First, does amplification represent an initial stage of immunodeficiency? We have detected an example of clonal amplification during primary infection (data not shown) suggesting clonal amplification can occur at any stage of infection. Given that clonal amplification was detected in equilibrated and compartmentalized populations, this also suggests that clonal amplification is not determined by the overall state of immunodeficiency. Second, what is the cellular source where this amplification occurs? At one extreme clonally amplified sequences could be the product of a single cell. This seems unlikely since the seminal tract can support very complex populations in the compartmentalized state consistent with many available target cells, and some of the clonally amplified populations have some population structure suggesting they are the result of multiple rounds of replication (supported by the longer BEAST estimates of TMRCA for some of these populations). The alternative is that clonal amplification occurs in a population of cells that are not infected by diverse viral genotypes. We suggest that either uninfected CD4+ T cells concentrate in specific sites, or are seeded by a single cell that then expands, until the focus of cells becomes infected with a single virus that spreads through this isolated population until the target cells are depleted. This would explain the self-limiting nature of the clonal amplification and explain how several clonal amplifications can occur concurrently. Finally, this process could be at work during compartmentalized virus replication, and thus account for the clonal amplification process also appearing during the replication of a complex compartmentalized population.

**Figure 6:**
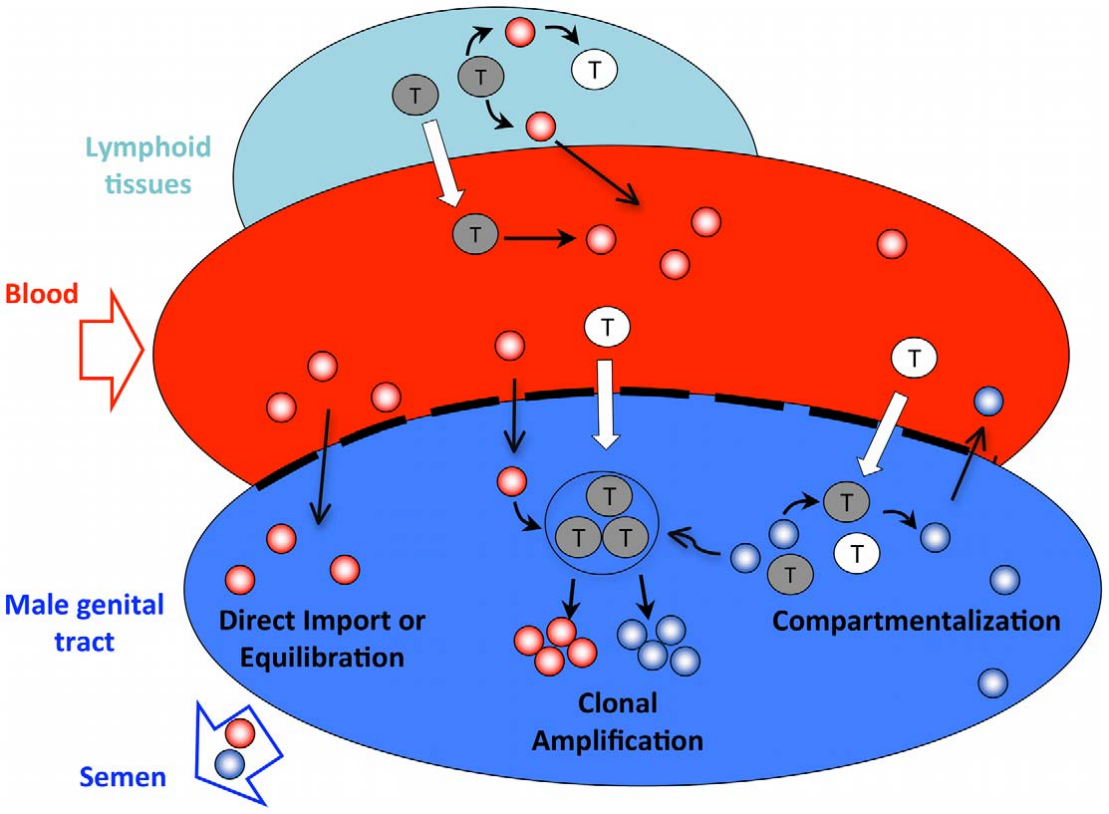
Model demonstrating HIV-1 populations in the blood and male genital tract. Target cells traffic from blood to the male genital tract. A semi-permeable barrier separates blood from the genital tract that allows passage of some free virus and uninfected and infected cells. Equilibration of blood and seminal plasma sequences occurs through direct import of sequences from blood to semen. Clonal amplification results from establishment of a local focus of infection of anatomically isolated uninfected cells in the genital tract. Compartmentalization occurs when resident cell populations in the male genital tract become infected and there is persistent local replication. Gray circle, HIV-1 infected target cell. White circle, HIV-1 uninfected target cell. Red circle, blood-derived virus. Blue circle, male genital tract-derived virus.

A corollary of the isolation of the clonally amplified population is that the complex, compartmentalized population must be sustained by a distinct mechanism. There is likely continued import of virus from blood; however, the amount of locally replicating virus must obscure detection of this imported population. Based on these inferences we propose a model (Fig. 6) to account for virus in semen. An assumption of this model is that viral populations within blood and semen are turning over similarly, and this is supported by a recent report in the literature showing similar decay kinetics of HIV-1 populations in blood and semen in men who initiate antiviral therapy [47]. In addition, our model is distinct from the semen being a viral reservoir, which is associated with reduced levels of viral replication [48]. We suggest that virus in the semen is derived from multiple sources. First, there is direct import of virus from the blood compartment, potentially without replication in the seminal tract, accounting for virus that is fully equilibrated between the blood and seminal tract compartments. Second, there is infiltration of individual infected CD4+ cells or virions into pockets of uninfected target cells that generate local foci of infection in the seminal tract, giving rise to clonal amplification of virus in this compartment. Third, ongoing local immune activation provides an environment that can support sustained, autonomous virus replication giving rise to compartmentalized virus. We estimate that this distinct population can replicate independently for a significant period of time, although lack of information about the rate of evolution in the compartment precludes a detailed analysis of the age of the population.

An alternative interpretation of the appearance of compartmentalization is that there is delayed equilibration between blood and the seminal tract. In this circumstance a change in the population in the blood would not immediately be reflected in the semen, giving the transient appearance of compartmentalization. We do not favor this interpretation since the complexity of the virus in the semen can be quite high giving TMRCA values of months to years. However, the analysis of longitudinal samples in subjects displaying compartmentalization will be required to resolve this issue.

An important unanswered question is the site within the seminal tract where virus undergoes independent replication. A relevant observation in this regard is that vasectomy does not preclude the presence of virus in semen [49], [50], suggesting that production of significant amounts of virus occurs outside of the testis, and implicating the seminal vesicles and prostate. Moreover, distal genitourinary sources other than the prostate have been implicated as the major source of seminal HIV-1 in men without urethritis or prostatitis [51]. In the setting of the blood compartment, disease progression is associated with higher levels of immune cell activation [52]. This may reflect an increasing trend to fail to control viral replication but with a continued response to the presence of viral antigen. We suggest a similar process may occur in the seminal tract and perhaps in other peripheral sites of viral replication. Recent literature reports the existence of clonal amplification of HIV-1 sequence in other compartments, including the CSF [53], breast milk [54], and cervicovaginal lavage fluid [55]. Thus, an influx of activated immune cells into areas where virus suppression is incomplete could lead to sustained viral replication and a distinct evolutionary pathway. In this regard, the presence of activated immune cell infiltrates that have been observed in the seminal tract of SIV-infected macaques [18] provides the likely sites where viral replication could occur in the male genital tract.

A result of independent replication in the seminal tract, both clonal amplification and sustained replication, is to alter the composition of the viral population in the semen relative to that in the blood plasma. These differences can make blood a suboptimal surrogate for the seminal compartment in assessing the relationship of virus in the donor and recipient of a sexual transmission event. Several studies have noted differences between the transmitted virus and the virus in donor blood for subtypes A, C, and D [56]–[60] but not subtype B [35], [58], with differences in either glycosylation patterns, variable loop lengths, or susceptibility to neutralizing antibodies. It will be important to determine if the distinctive features of virus in semen play a role in transmission and/or in defining the nature of the transmitted virus.

## Materials and Methods

### Ethics statement and source of clinical samples

The patients infected with HIV-1 subtype C (n = 12) were enrolled through the Kamuzu Central Hospital in Lilongwe, Malawi, between January and March, 1996 [33]. The protocol was approved by the University of North Carolina Committee on the Protection of Human Rights and the Malawi Health Sciences Research Committee. All study participants gave written informed consent and were offered a small payment for their participation. The original study design was a prospective, sequential comparison of two cohorts: HIV-1-infected men with urethritis who had urethral discharge on physical exam and at least five white blood cells per high-power field from a urethral swab, selected from the STI clinic, and HIV-infected men without urethritis on physical exam, selected from the dermatology clinic [33]. Blood and semen samples used in the current study were collected from men attending the dermatology clinic in Lilongwe, Malawi as described previously [33]. For both the STI and dermatology clinics, screening for gonorrhea, trichomonas, syphilis, and chlamydia was performed. In addition, blood and semen samples were obtained from participants with HIV-1 subtype B from the US (n = 4) who were enrolled through the CHAVI 001 clinical core, a multi-center, prospective, observational cohort study of acute HIV-1 infection. IRB approval was awarded by each participating center as well as the Division of AIDS. All study participants gave written informed consent and were offered a small payment for their participation. None of the subtype B infected participants had urethritis on physical exam, and were negative for gonorrhea, Chlamydia, syphilis, and trichomonas infection. Consistent with established infection, all HIV-1 infected Malawi and US patients were confirmed EIA and Western Blot positive at study enrollment. Paired blood and seminal plasma samples from HIV-1 uninfected males for cytokine/chemokine analyses were obtained from the CHAVI 001 clinical core sites in the US (n = 12) and Africa (n = 6).

### Extraction of viral RNA and generation of amplicons

Cell-free blood plasma and seminal plasma were isolated and frozen as previously described [1]. HIV-1 viral loads from blood and seminal plasma from the Malawi men were determined by quantitative nucleic-acid sequence-based-analysis (NASBA, Organon-Teknika) [33], and by Roche Amplicor vRNA or Abbott RealTime HIV-1 assays for the US men. Virus in the seminal plasma was pelleted by centrifugation prior to RNA isolation to remove the seminal plasma. The blood plasma or the resuspended virus pellet from the seminal plasma was extracted to isolate viral RNA using the QIAMP Viral RNA Mini Kit (Qiagen). For each sample, approximately 10,000 viral RNA copies based on viral load were extracted and eluted. cDNA synthesis was performed using Superscript III Reverse Transcriptase (Invitrogen) with an oligo-d(T) primer as previously described [34]–[36]. To confirm that proviral DNA was not the source of SGA *env*-derived amplicons from cell-free viral RNA, RT-minus blood (n = 11) and seminal (n = 11) plasma samples were subjected to the SGA protocol; the remaining samples had insufficient volume remaining for the RT-minus control experiment. To preclude PCR recombination and Taq-induced errors, single genome amplification (SGA) of the *env* gene was performed using limiting dilution [34]–[36], [61]–[63]. PCR amplicons were bidirectionally sequenced. To ensure that sequences arose from single DNA molecules, chromatograms with double peaks, indicating amplification from more than one cDNA template, were excluded. SGA-derived *env* amplicons with frameshift mutations that resulted in premature stop codons were also excluded. GenBank accession numbers are HM638460 to HM639260.

### Analysis of viral sequences

DNA sequence alignments were performed using clustal W [64]. Phylogenetic trees were generated using a neighbor-joining method (MEGA 4.0) [65]. Pairwise DNA distances were computed using MEGA 4.0. Highlighter plots were generated to visualize sequence differences (www.hiv.lanl.gov). A Tajima's D test for neutrality was performed for each patient using DnaSP [43]. Compartmentalization of viral sequences was assessed by using the Slatkin-Maddison test [37] and correlation coefficient [38] available through HyPhy [39]. Gene flow was determined by the number of migration events compared between semen and blood after 10,000 permutations for the Slatkin-Maddison test. Compartmentalization was defined when P values <0.01 were obtained with the Slatkin-Maddison test using all sequences except the clonally-amplified sequences, of which only one was included, and when concordant results were obtained with the correlation coefficient test. More extreme P values were obtained when all of the clonally amplified sequences were included (Supplemental Table S2).

### Detection of cytokines

Nineteen cytokines and chemokines were analyzed by luminex from paired blood and seminal plasma from 12 HIV-1 infected and uninfected subjects as previously described [66]. Concentrations of IL-1b, IL-2, IL-4, IL-5, IL-6, IL-7, IL-8, IL-10, IL-12 (p70), IL-13, IFN-g, TNF-a, and GM-CSF were measured using LINCOplex Luminex high-sensitivity 13-plex kits (Millipore) according to the manufacturer's instructions. Concentrations of MIP-1a, MIP-1b, RANTES, MCP-1, MIG, and IP-10 were measured using custom standard sensitivity 6-plex kits (Bio-Rad) according to the manufacturer's instructions. Each sample was assayed in duplicate, and cytokine standards supplied by the manufacturer were run in parallel. Data were collected using the Bio-plex Suspension Array Reader (Bio-Rad) and a regression formula was used to calculate sample concentrations from standard curves. Values that were below the lower limit of detection were reported as the mid-point between the lower level of detection and zero. Semen:blood analyte ratios were calculated for each subject; however, data were excluded if both compartments were below the level of detection. Saturated values were reported as the upper limit of detection. Sensitivity values were adjusted for samples where the volume was limited and had to be diluted before the measurement. Statistical tests comparing analyte levels were non-parametric Mann-Whitney-U test for two groups (compartmentalization vs. equilibration), and Kruskal-Wallis test for three groups (high amplification, low amplification, no amplification). The non-parametric Mann-Whitney test was used to compare semen:blood cytokine ratios between HIV-1 infected and uninfected subjects.

### Bayesian analysis

For each subject, blood and semen sequences were aligned using ClustalW 2.0.7 [67]. Markov Chain Monte Carlo Simulation (MCMC) using Bayesian inference was used to resolve a phylogenetic tree with the highest posterior probability to estimate the time of divergence from the most recent common ancestor (MRCA) implemented in BEAST (Bayesian Evolutionary Analysis by Sampling Trees v.1.4.8) [41]. Each independent run had a chain length of 30,000,000 with a sample frequency of 1000. A general time-reversible substitution model was used, with site heterogeneity using a gamma distribution with a proportion of invariant sites and sampling across four categories. Analyses were performed using an HIV-1 generation time of 1.6 days [68]. Rate heterogeneity across codon positions was unlinked, and the mean fixed substitution rate was 2.16×10^−5^ under a relaxed uncorrelated exponential molecular clock. A coalescent piecewise-constant Bayesian Skyline model with ten groups was used as the tree prior. The MCMC log output of each run was examined in Tracer 1.4 to verify adequate chain mixing and estimated sample sizes of greater than 200 for parameters of interest, and log and tree files with a minimum of two independent runs were combined with a 10% burn-in using LogCombiner 1.4.8. The target tree for each patient was summarized using TreeAnnotater 1.4.8, and visualized in FigTree1.1.2.

## Supporting Information

Figure S1 Neighbor-joining tree of SGA-derived *env* amplicons for three HIV-1 subtype C patients demonstrating equilibration between blood and semen. Blood SGA-*env* sequences (red circles) and semen SGA-*env* sequences (blue triangles). Vertical blue bars highlight clonal amplification of specific variants. Bootstrap values ≥70 are shown. An outgroup was included to root the tree but is not shown.
(1.00 MB TIF)

Figure S2 Neighbor-joining tree of SGA-derived *env* amplicons for two HIV-1 subtype C patients demonstrating compartmentalization of seminal sequences. Blood SGA-*env* sequences (red circles) and semen SGA-*env* sequences (blue triangles). Ovals highlight clades of compartmentalized sequences in the semen or blood. Vertical blue bars highlight clonal amplification of specific variants. Bootstrap values ≥70 are shown. An outgroup was included to root the tree but is not shown.
(0.93 MB TIF)

Figure S3 Neighbor-joining tree of SGA-derived *env* amplicons for two HIV-1 subtype B patients demonstrating clonal amplification of seminal variants. Blood SGA-*env* sequences (red circles) and semen SGA-*env* sequences (blue triangles). Vertical blue bars highlight clonal amplification of specific variants. Bootstrap values ≥70 are shown. An outgroup was included to root the tree but is not shown.
(0.85 MB TIF)

Figure S4 Highlighter plot for patient C007 with clonal amplification in the seminal tract. All blood (B) and semen (S) sequences are included. A representative amplified seminal variant is used as the master to illustrate identical sequences within the seminal plasma. Each vertical tic represents a mismatch from the master sequence as outlined in the figure.
(3.81 MB TIF)

Figure S5 Highlighter plot for patient C009 with clonal amplification in the seminal tract. All blood (B) and semen (S) sequences are included. A representative amplified seminal variant is used as the master to illustrate identical sequences within the seminal plasma. Each vertical tic represents a mismatch from the master sequence as outlined in the figure.
(4.55 MB TIF)

Figure S6 Highlighter plot for patient C012 with clonal amplification in the seminal tract. All blood (B) and semen (S) sequences are included. A representative amplified seminal variant is used as the master to illustrate identical sequences within the seminal plasma. Each vertical tic represents a mismatch from the master sequence as outlined in the figure.
(3.88 MB TIF)

Figure S7 Highlighter plot for patient C019 with clonal amplification in the seminal tract. All blood (B) and semen (S) sequences are included. A representative amplified seminal variant is used as the master to illustrate identical sequences within the seminal plasma. Each vertical tic represents a mismatch from the master sequence as outlined in the figure.
(2.62 MB TIF)

Figure S8 Highlighter plot for patient C047 with clonal amplification in the seminal tract. All blood and semen (S) sequences are included. A representative amplified seminal variant is used as the master to illustrate identical sequences within the seminal plasma. Each vertical tic represents a mismatch from the master sequence as outlined in the figure.
(2.81 MB TIF)

Figure S9 Highlighter plot for patient C070 with clonal amplification in the seminal tract. All blood (B) and semen (S) sequences are included. A representative amplified seminal variant is used as the master to illustrate identical sequences within the seminal plasma. Each vertical tic represents a mismatch from the master sequence as outlined in the figure.
(3.99 MB TIF)

Figure S10 Highlighter plot for patient C109 with clonal amplification in the seminal tract. All blood (B) and semen (S) sequences are included. A representative amplified seminal variant is used as the master to illustrate identical sequences within the seminal plasma. Each vertical tic represents a mismatch from the master sequence as outlined in the figure.
(2.01 MB TIF)

Figure S11 Highlighter plot for patient C113 with clonal amplification in the seminal tract. All blood (B) and semen (S) sequences are included. A representative amplified seminal variant is used as the master to illustrate identical sequences within the seminal plasma. Each vertical tic represents a mismatch from the master sequence as outlined in the figure.
(3.26 MB TIF)

Figure S12 Highlighter plot for patient 700010333 with clonal amplification in the seminal tract. All blood (B) and semen (S) sequences are included. A representative amplified seminal variant is used as the master to illustrate identical sequences within the seminal plasma. Each vertical tic represents a mismatch from the master sequence as outlined in the figure.
(3.71 MB TIF)

Figure S13 Highlighter plot for patient 700010501 with clonal amplification in the seminal tract. All blood (B) and semen (S) sequences are included. A representative amplified seminal variant is used as the master to illustrate identical sequences within the seminal plasma. Each vertical tic represents a mismatch from the master sequence as outlined in the figure.
(2.56 MB TIF)

Figure S14 Highlighter plot for patient 700011145 with clonal amplification in the seminal tract. All blood (B) and semen (S) sequences are included. A representative amplified seminal variant is used as the master to illustrate identical sequences within the seminal plasma. Each vertical tic represents a mismatch from the master sequence as outlined in the figure.
(2.27 MB TIF)

Figure S15 Highlighter plot for patient 701010380 with clonal amplification in the seminal tract. All blood (B) and semen (S) sequences are included. A representative amplified seminal variant is used as the master to illustrate identical sequences within the seminal plasma. Each vertical tic represents a mismatch from the master sequence as outlined in the figure.
(4.05 MB TIF)

Figure S16 Relationship between documented time post-seroconversion in years for patient 11 plasma (42) and BEAST estimated time post-infection in years. Longitudinal samples were analyzed using BEAST as outlined in the Methods.
(0.12 MB TIF)

Table S1 Semen and blood cytokine/chemokine analytes (pg/ml) and ratios for HIV-1 infected and uninfected men.
(0.08 MB XLS)

Table S2 Results of Slatkin-Maddison test for compartmentalization using all blood and semen derived sequences, and with each amplified variant only once.
(0.03 MB XLS)
