# Supplementary figures and images for: HIV-1 Populations in Semen Arise through Multiple Mechanisms
Source: PLoS Pathog. 2010 Aug 19;6(8):e1001053. doi: 10.1371/journal.ppat.1001053 (PMC2924360; doi:10.1371/journal.ppat.1001053)

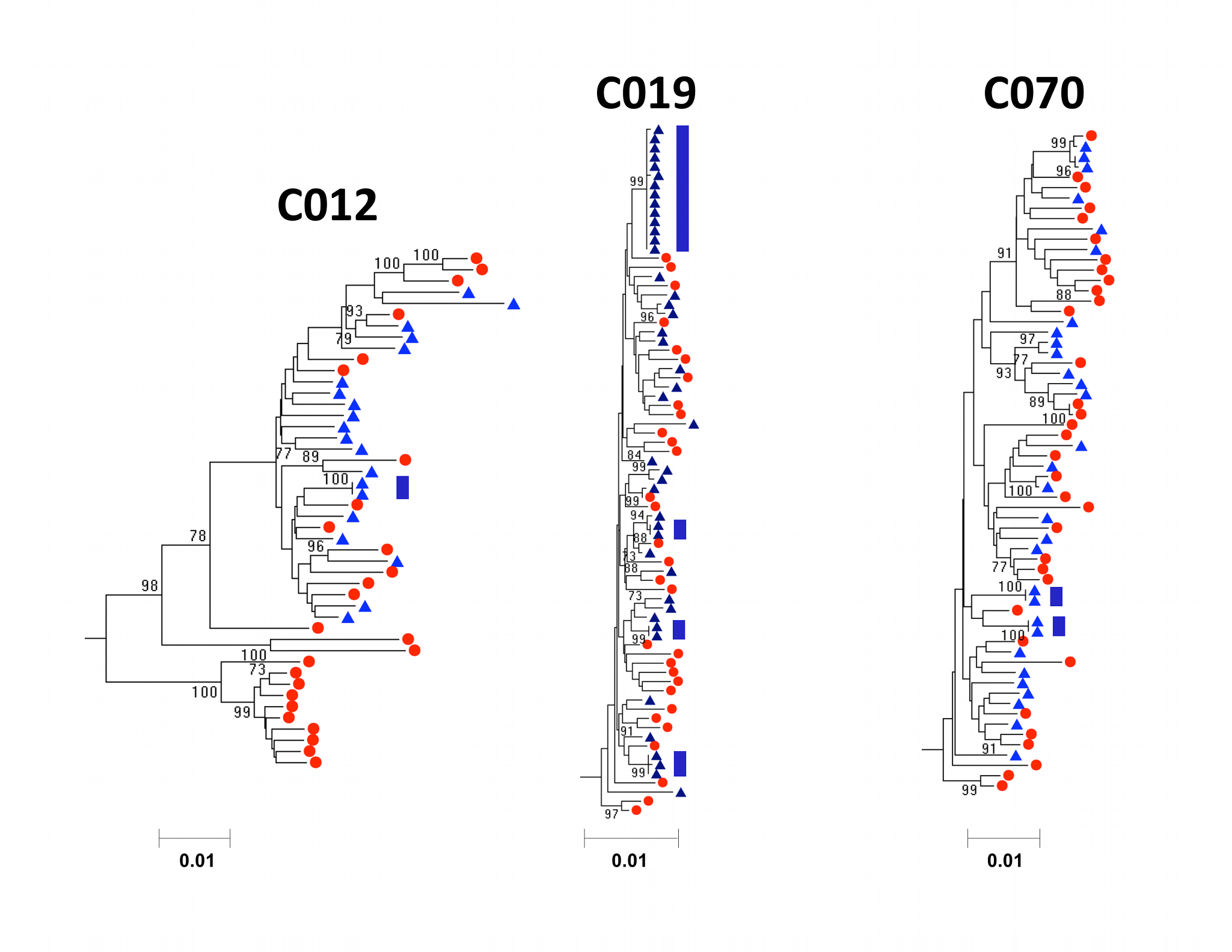

Supplement: Figure S1 — Neighbor-joining tree of SGA-derived env amplicons for three HIV-1 subtype C patients demonstrating equilibration between blood and semen. Blood SGA-env sequences (red circles) and semen SGA-env sequences (blue triangles). Vertical blue bars highlight clonal amplification of specific variants. Bootstrap values ≥70 are shown. An outgroup was included to root the tree but is not shown. (1.00 MB TIF) [file ppat.1001053.s001.tif]

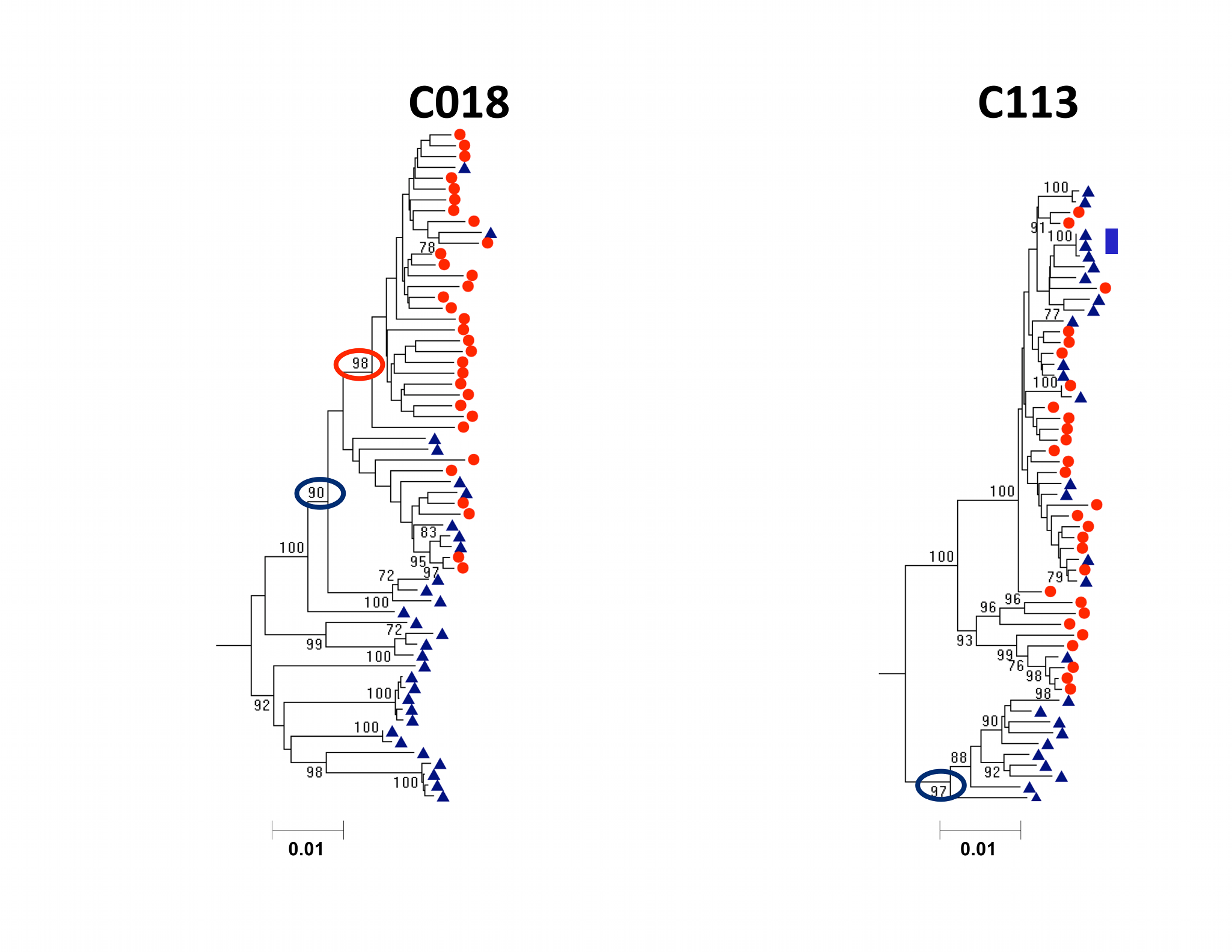

Supplement: Figure S2 — Neighbor-joining tree of SGA-derived env amplicons for two HIV-1 subtype C patients demonstrating compartmentalization of seminal sequences. Blood SGA-env sequences (red circles) and semen SGA-env sequences (blue triangles). Ovals highlight clades of compartmentalized sequences in the semen or blood. Vertical blue bars highlight clonal amplification of specific variants. Bootstrap values ≥70 are shown. An outgroup was included to root the tree but is not shown. (0.93 MB TIF) [file ppat.1001053.s002.tif]

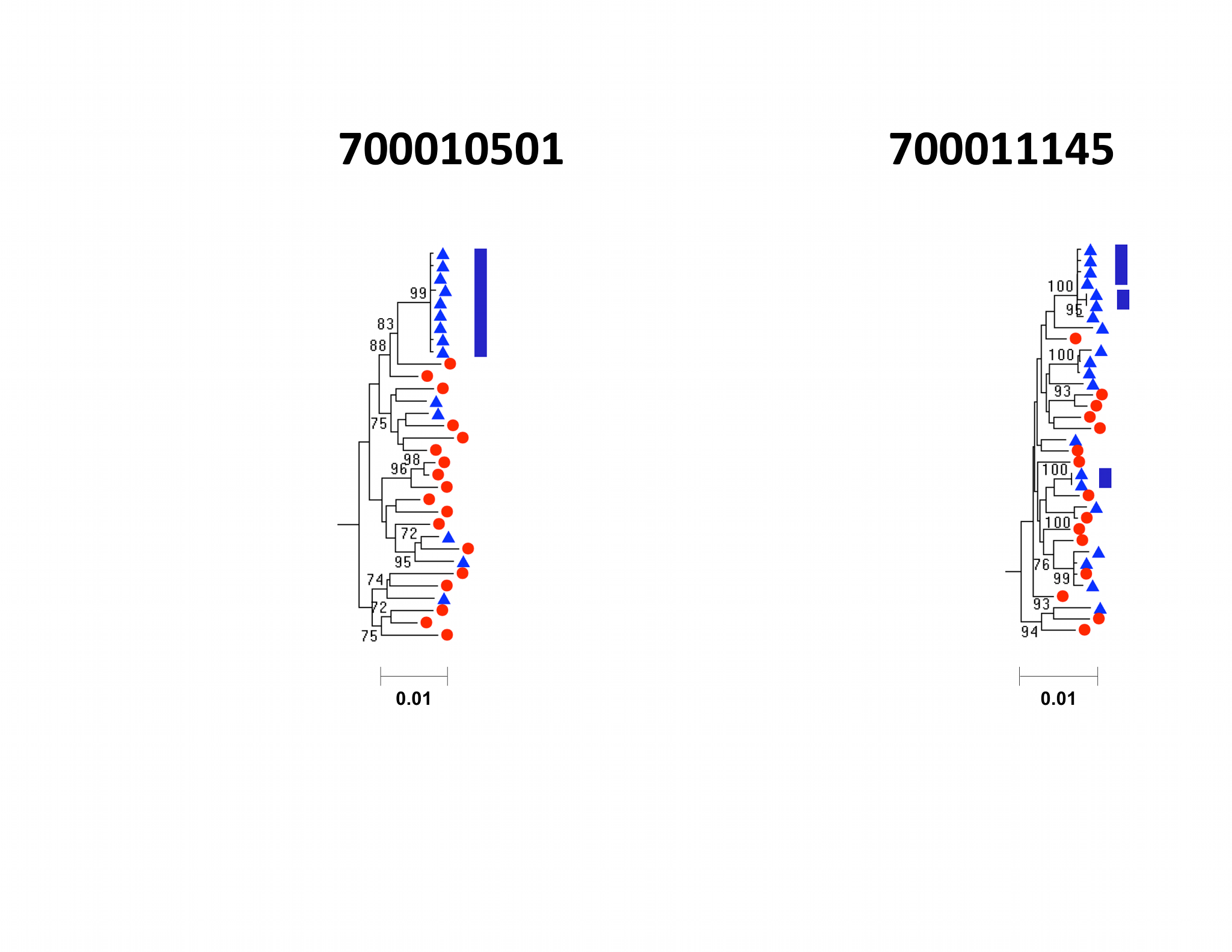

Supplement: Figure S3 — Neighbor-joining tree of SGA-derived env amplicons for two HIV-1 subtype B patients demonstrating clonal amplification of seminal variants. Blood SGA-env sequences (red circles) and semen SGA-env sequences (blue triangles). Vertical blue bars highlight clonal amplification of specific variants. Bootstrap values ≥70 are shown. An outgroup was included to root the tree but is not shown. (0.85 MB TIF) [file ppat.1001053.s003.tif]

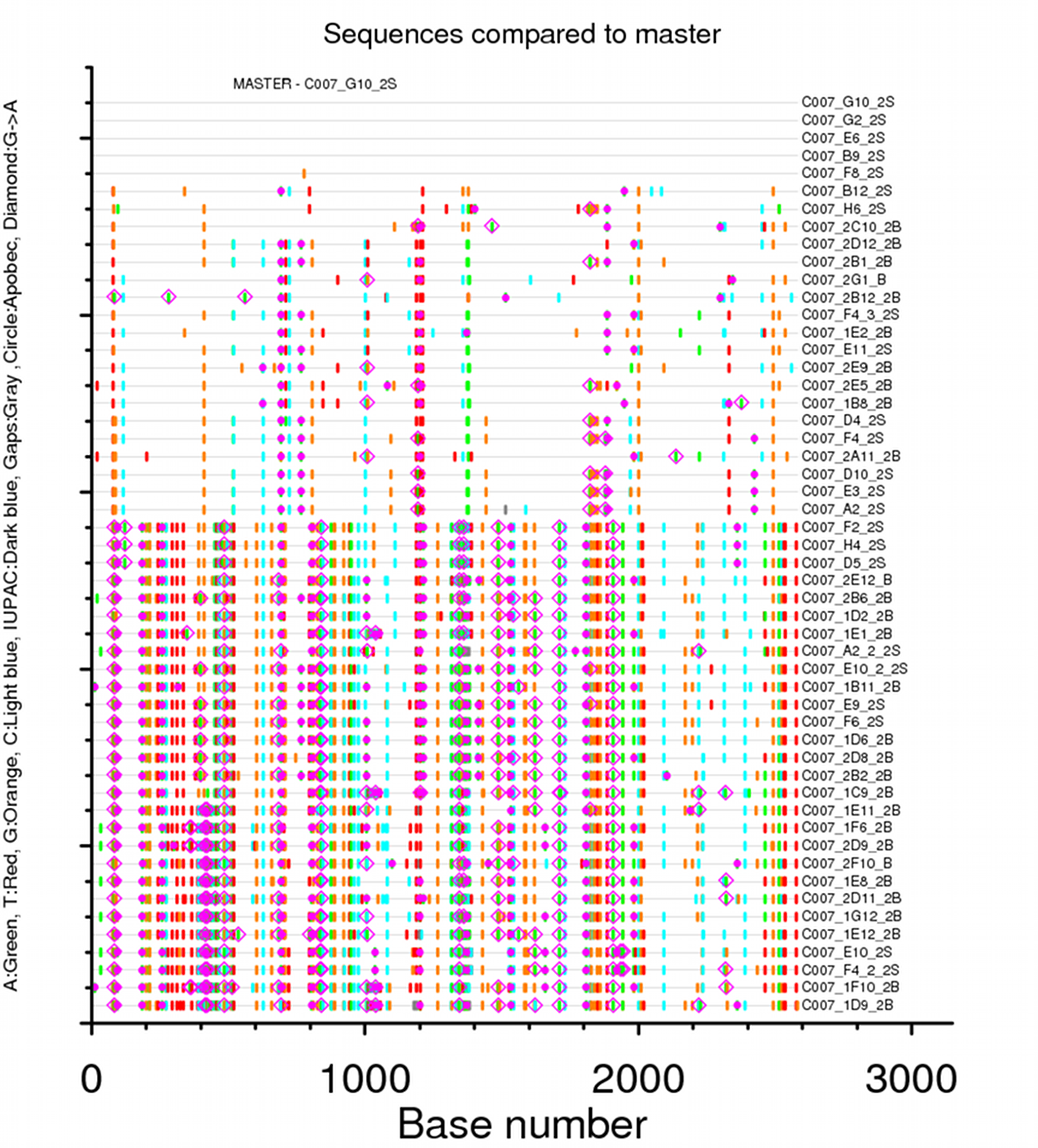

Supplement: Figure S4 — Highlighter plot for patient C007 with clonal amplification in the seminal tract. All blood (B) and semen (S) sequences are included. A representative amplified seminal variant is used as the master to illustrate identical sequences within the seminal plasma. Each vertical tic represents a mismatch from the master sequence as outlined in the figure. (3.81 MB TIF) [file ppat.1001053.s004.tif]

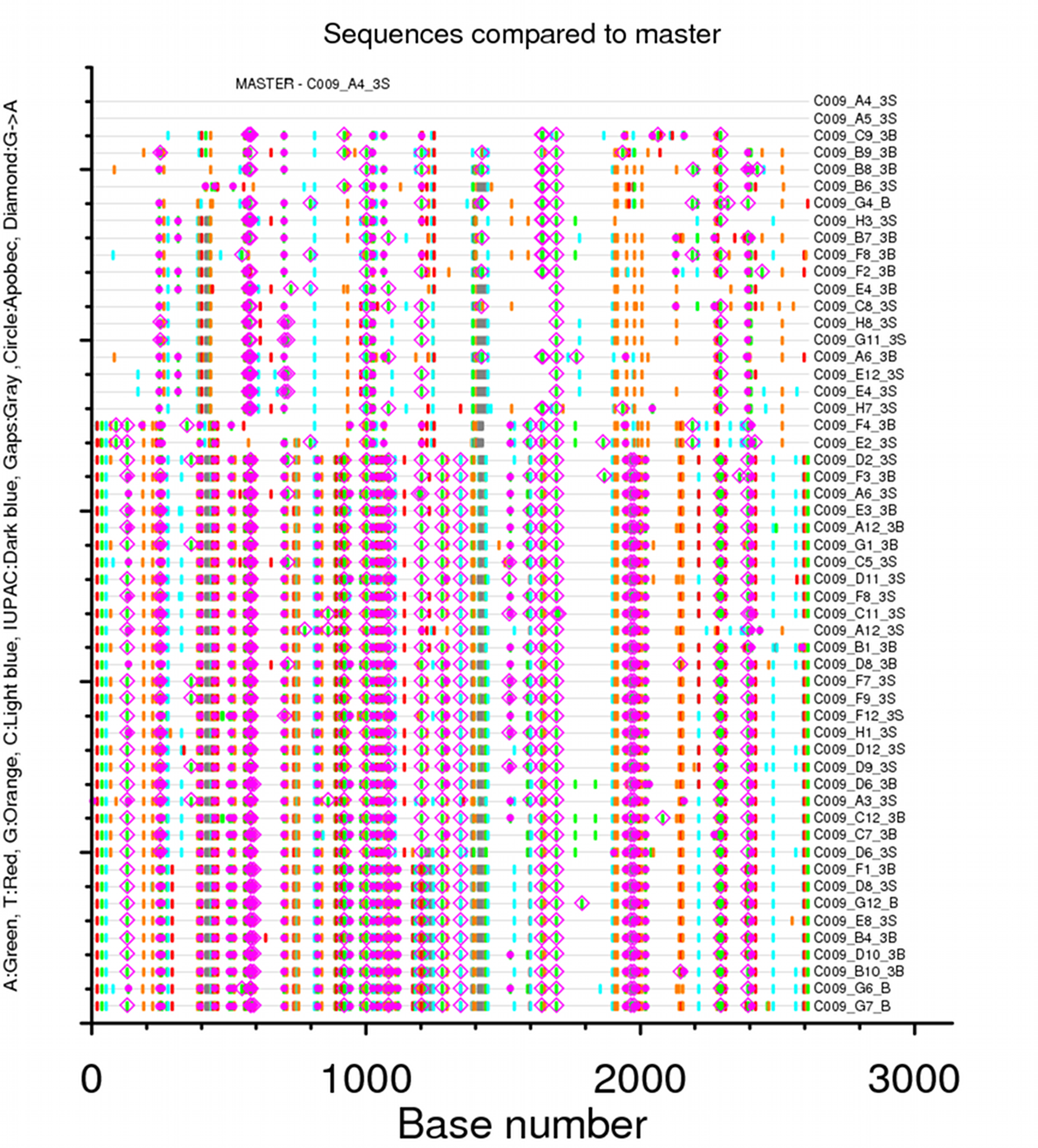

Supplement: Figure S5 — Highlighter plot for patient C009 with clonal amplification in the seminal tract. All blood (B) and semen (S) sequences are included. A representative amplified seminal variant is used as the master to illustrate identical sequences within the seminal plasma. Each vertical tic represents a mismatch from the master sequence as outlined in the figure. (4.55 MB TIF) [file ppat.1001053.s005.tif]

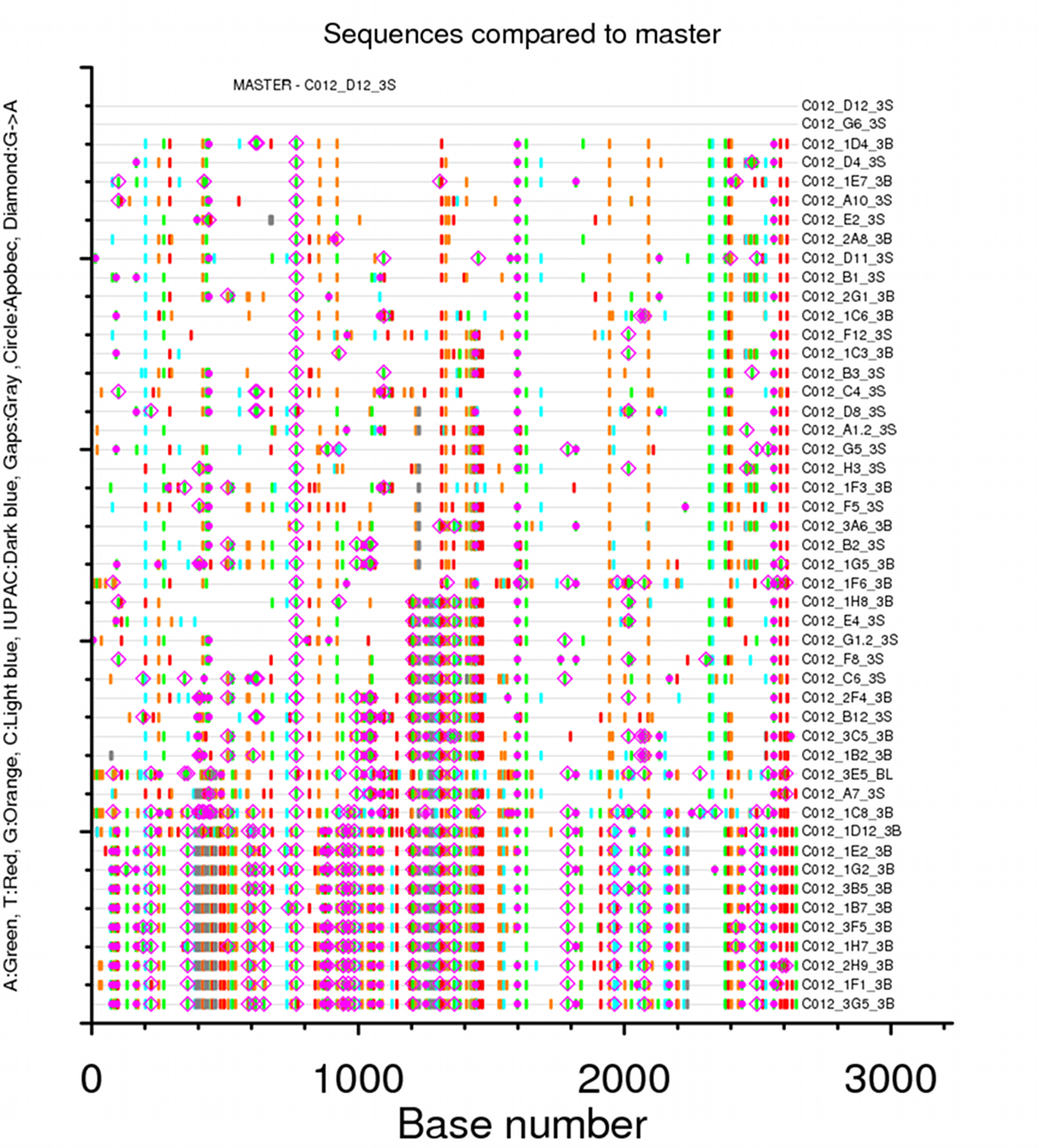

Supplement: Figure S6 — Highlighter plot for patient C012 with clonal amplification in the seminal tract. All blood (B) and semen (S) sequences are included. A representative amplified seminal variant is used as the master to illustrate identical sequences within the seminal plasma. Each vertical tic represents a mismatch from the master sequence as outlined in the figure. (3.88 MB TIF) [file ppat.1001053.s006.tif]

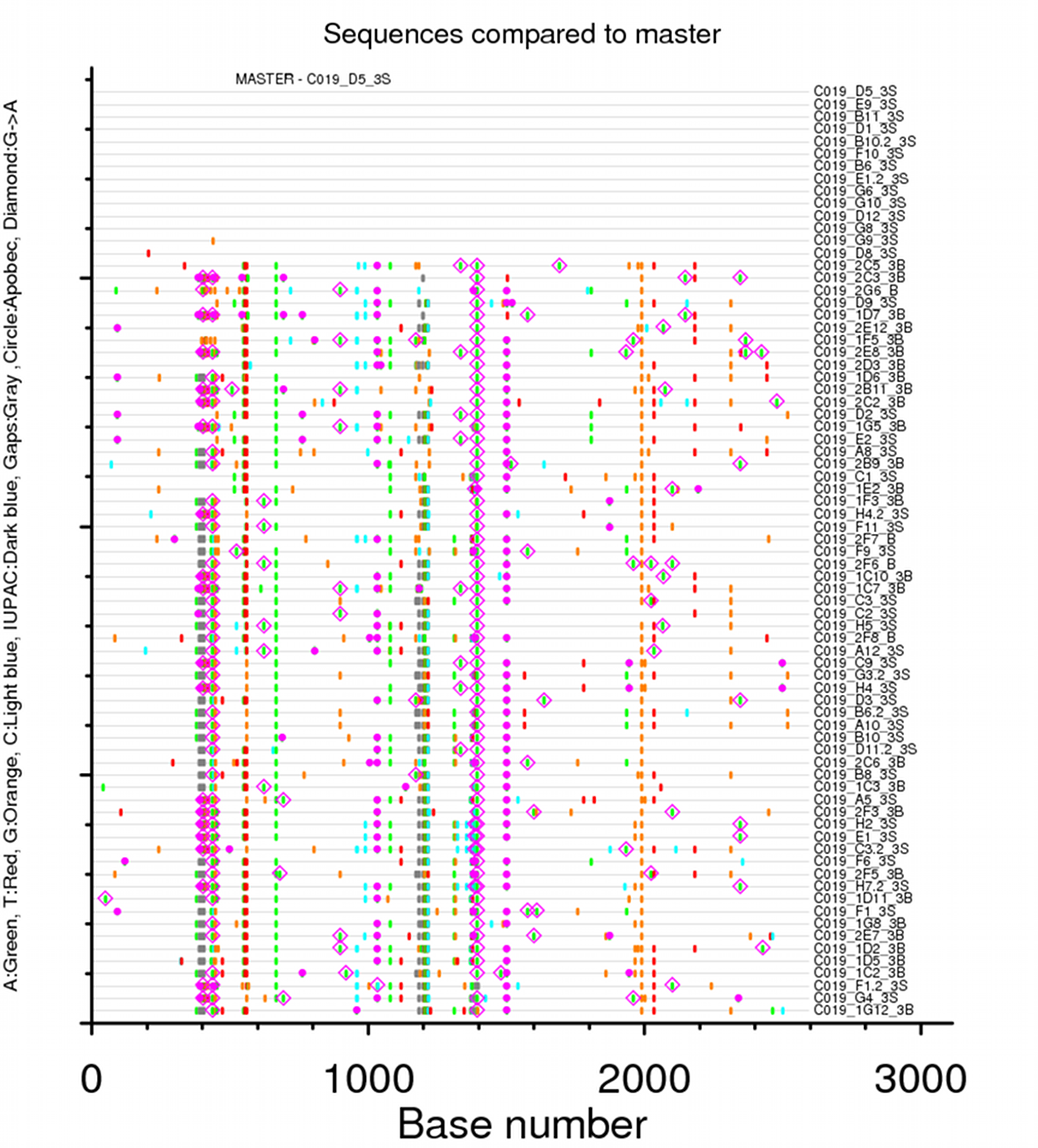

Supplement: Figure S7 — Highlighter plot for patient C019 with clonal amplification in the seminal tract. All blood (B) and semen (S) sequences are included. A representative amplified seminal variant is used as the master to illustrate identical sequences within the seminal plasma. Each vertical tic represents a mismatch from the master sequence as outlined in the figure. (2.62 MB TIF) [file ppat.1001053.s007.tif]

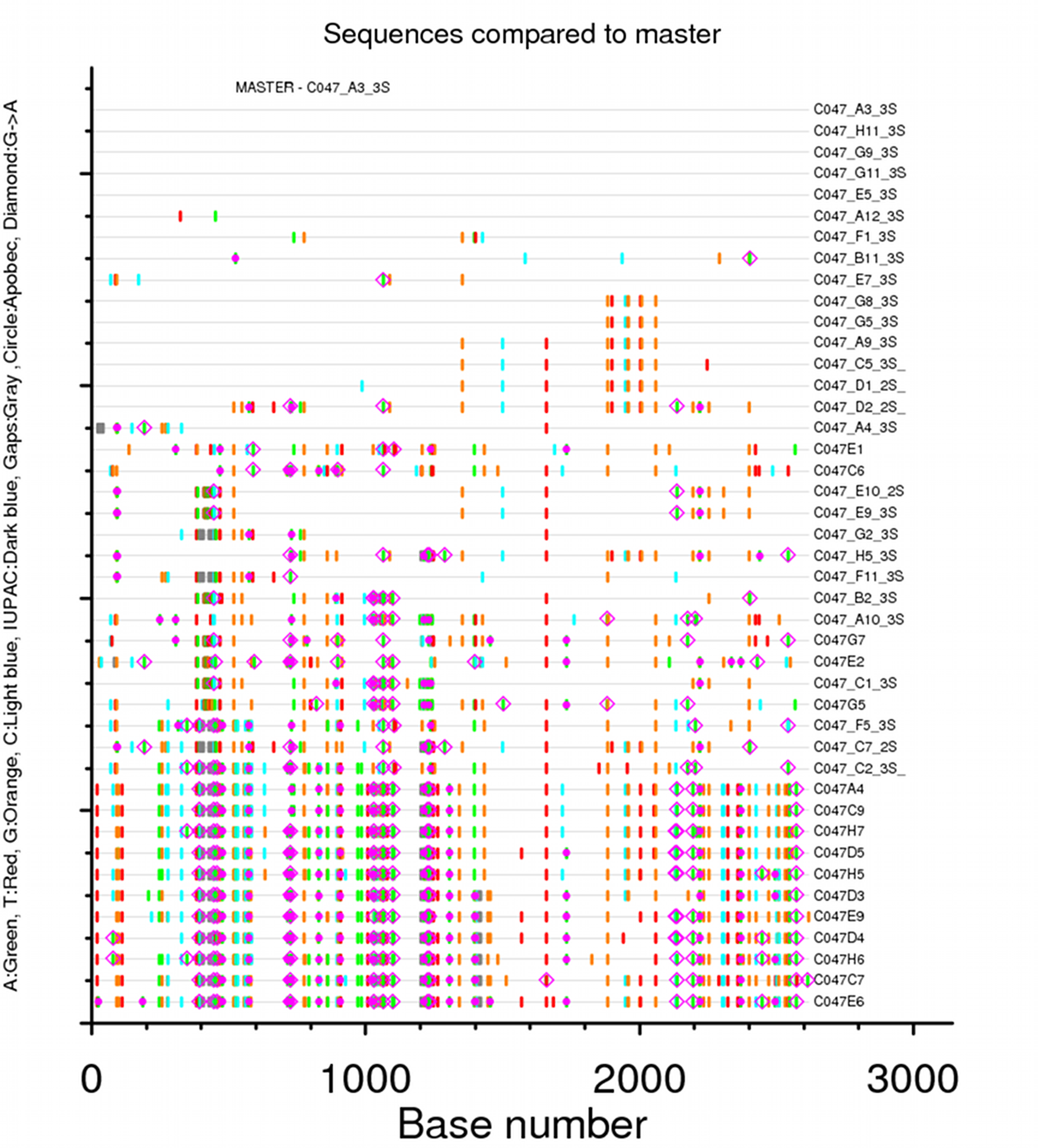

Supplement: Figure S8 — Highlighter plot for patient C047 with clonal amplification in the seminal tract. All blood and semen (S) sequences are included. A representative amplified seminal variant is used as the master to illustrate identical sequences within the seminal plasma. Each vertical tic represents a mismatch from the master sequence as outlined in the figure. (2.81 MB TIF) [file ppat.1001053.s008.tif]

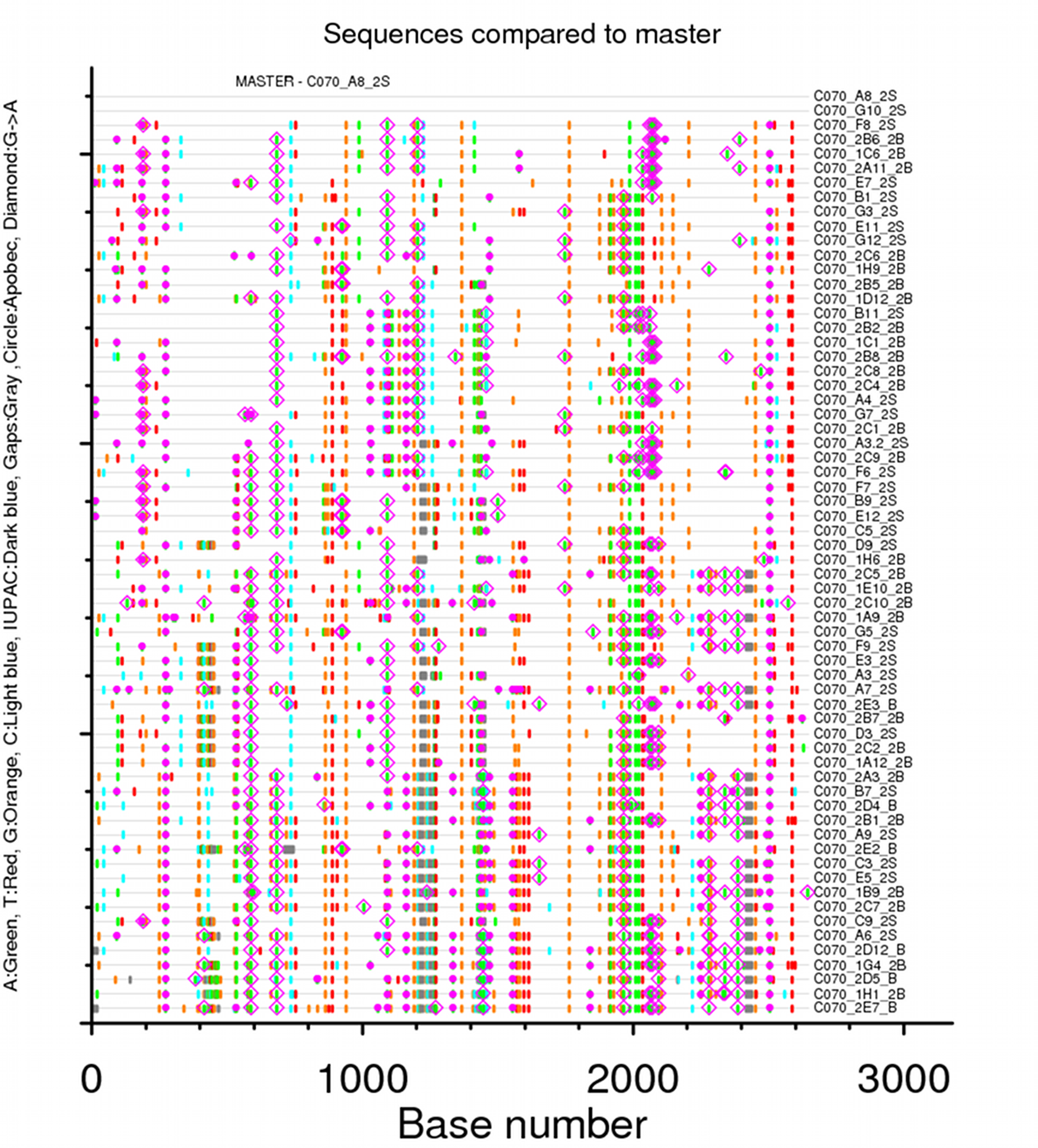

Supplement: Figure S9 — Highlighter plot for patient C070 with clonal amplification in the seminal tract. All blood (B) and semen (S) sequences are included. A representative amplified seminal variant is used as the master to illustrate identical sequences within the seminal plasma. Each vertical tic represents a mismatch from the master sequence as outlined in the figure. (3.99 MB TIF) [file ppat.1001053.s009.tif]

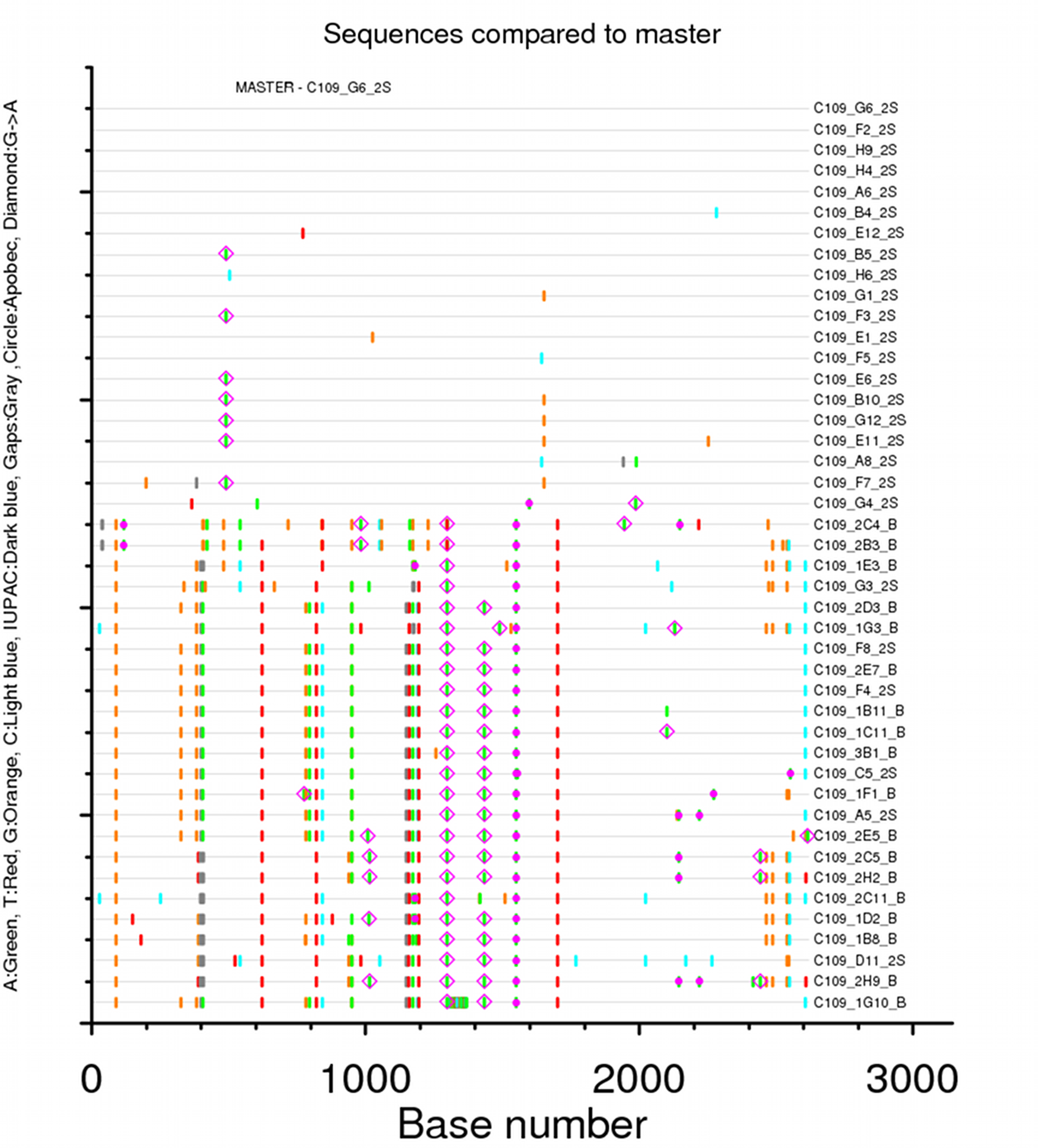

Supplement: Figure S10 — Highlighter plot for patient C109 with clonal amplification in the seminal tract. All blood (B) and semen (S) sequences are included. A representative amplified seminal variant is used as the master to illustrate identical sequences within the seminal plasma. Each vertical tic represents a mismatch from the master sequence as outlined in the figure. (2.01 MB TIF) [file ppat.1001053.s010.tif]

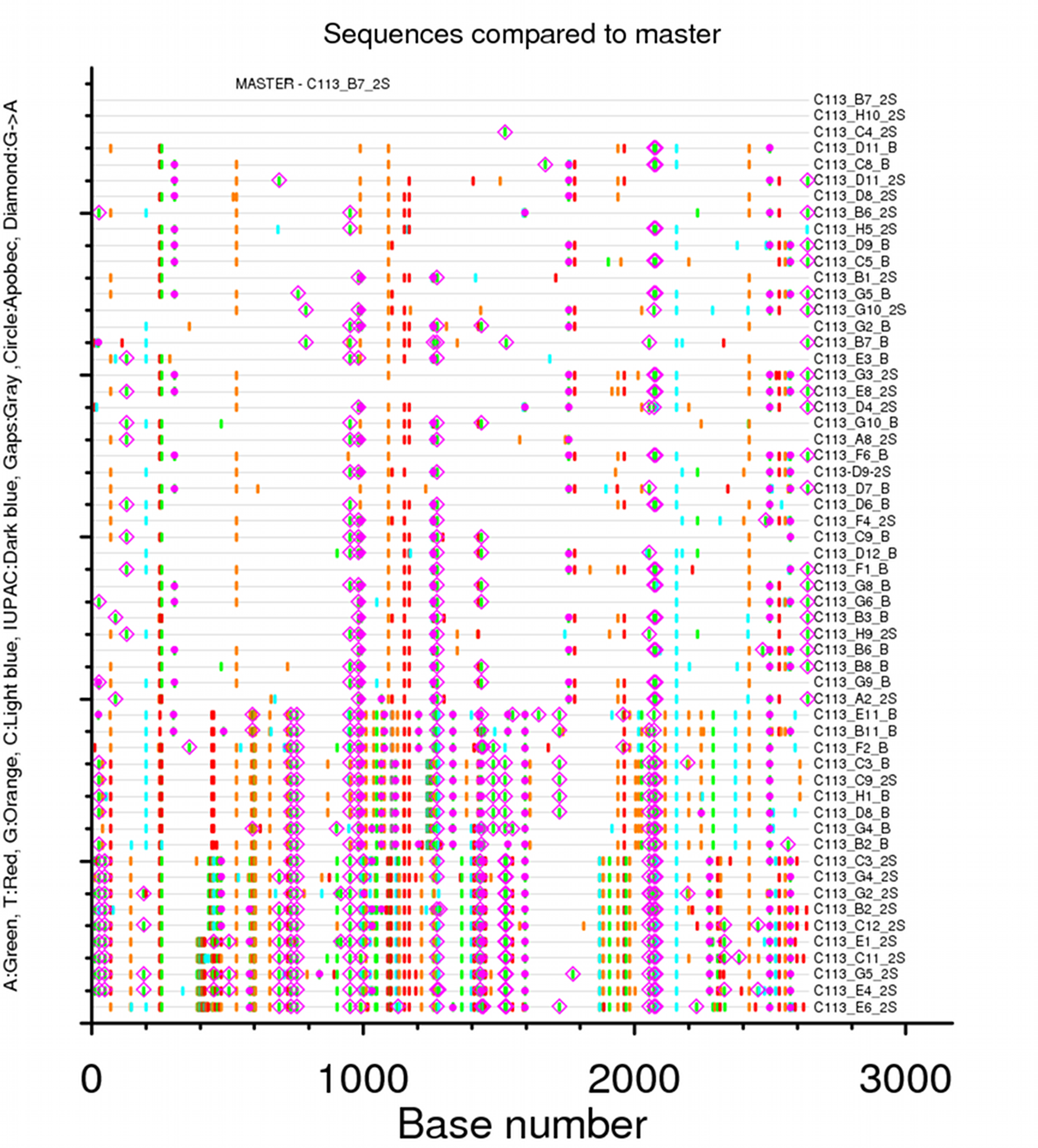

Supplement: Figure S11 — Highlighter plot for patient C113 with clonal amplification in the seminal tract. All blood (B) and semen (S) sequences are included. A representative amplified seminal variant is used as the master to illustrate identical sequences within the seminal plasma. Each vertical tic represents a mismatch from the master sequence as outlined in the figure. (3.26 MB TIF) [file ppat.1001053.s011.tif]

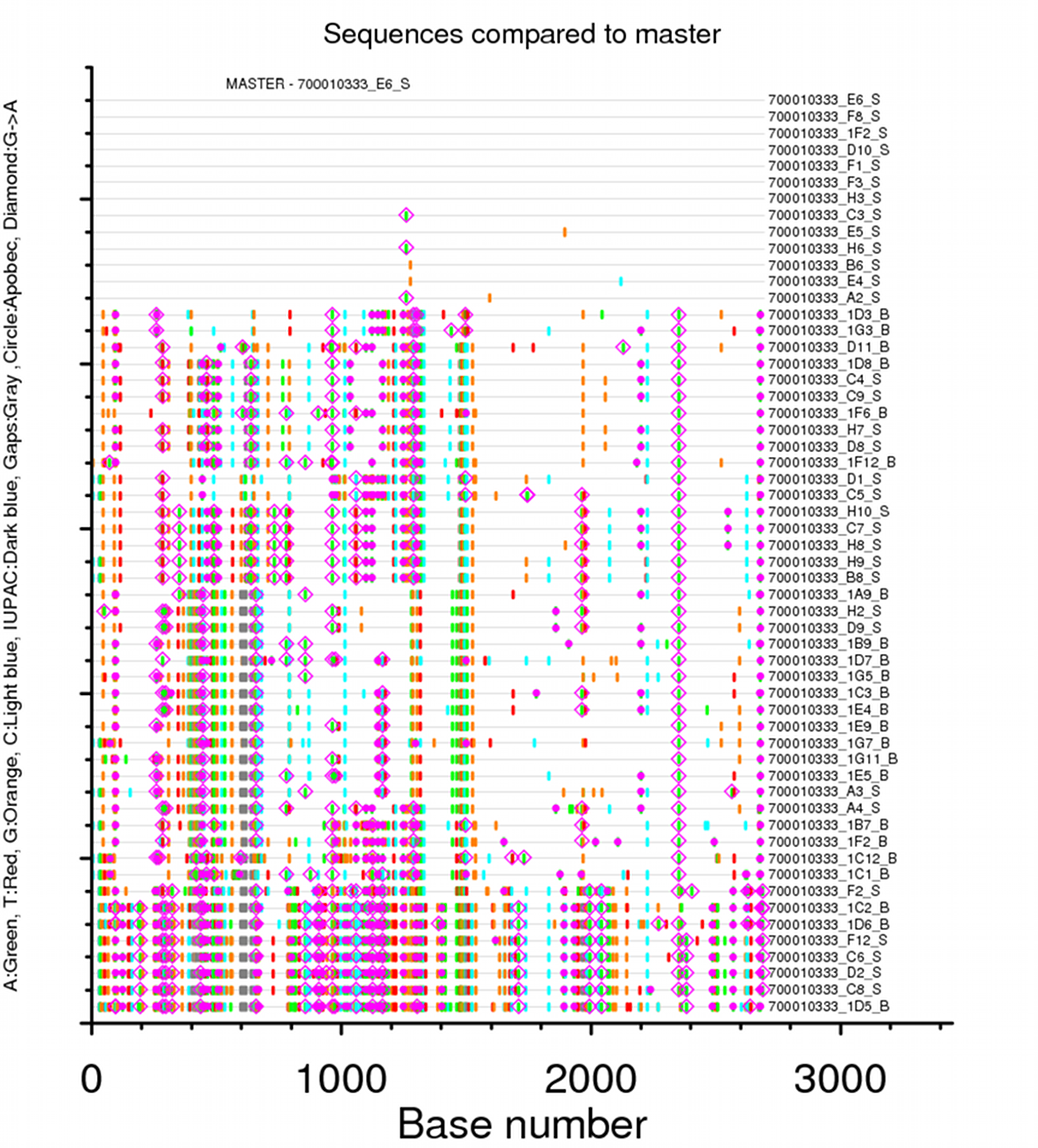

Supplement: Figure S12 — Highlighter plot for patient 700010333 with clonal amplification in the seminal tract. All blood (B) and semen (S) sequences are included. A representative amplified seminal variant is used as the master to illustrate identical sequences within the seminal plasma. Each vertical tic represents a mismatch from the master sequence as outlined in the figure. (3.71 MB TIF) [file ppat.1001053.s012.tif]

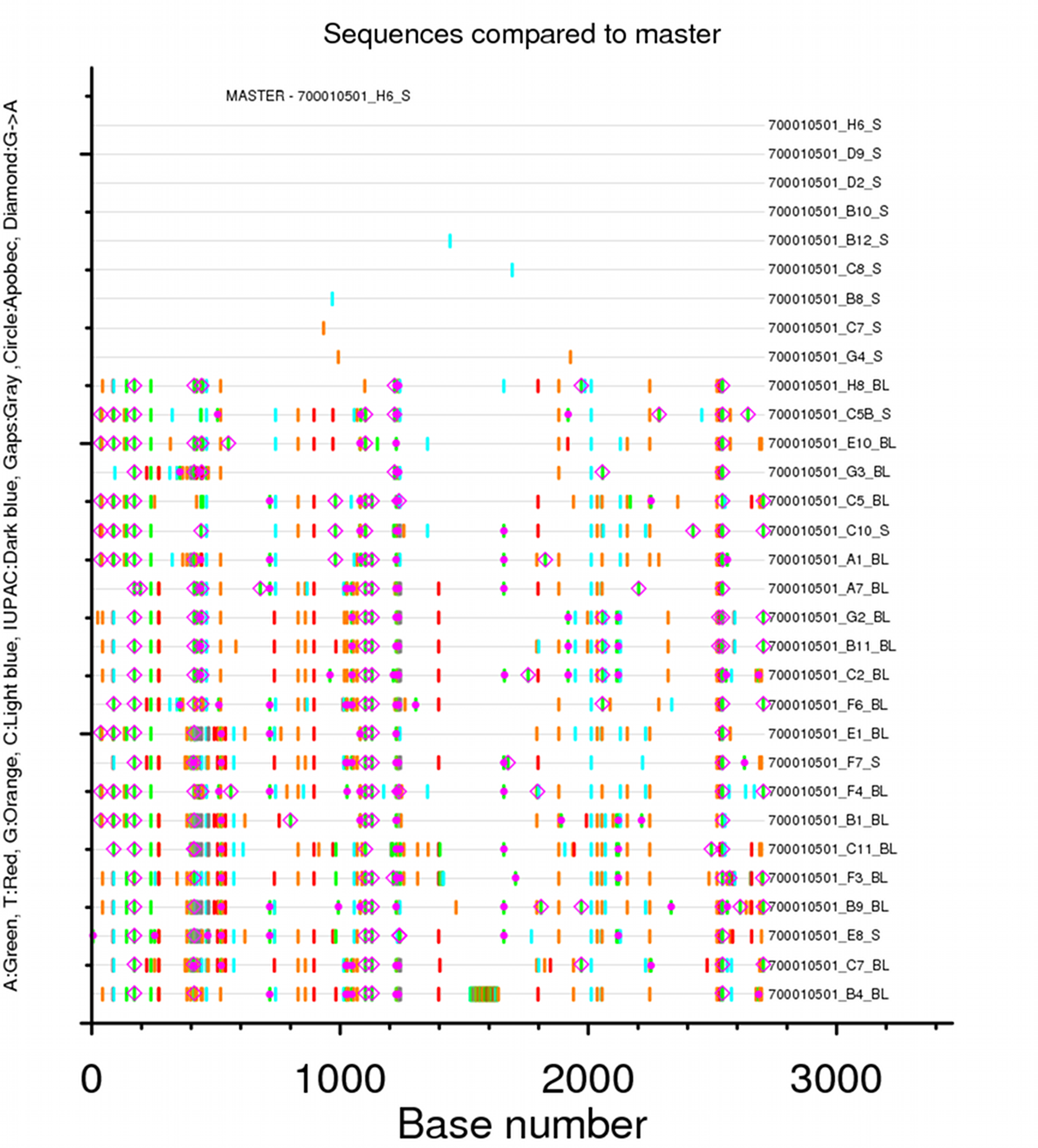

Supplement: Figure S13 — Highlighter plot for patient 700010501 with clonal amplification in the seminal tract. All blood (B) and semen (S) sequences are included. A representative amplified seminal variant is used as the master to illustrate identical sequences within the seminal plasma. Each vertical tic represents a mismatch from the master sequence as outlined in the figure. (2.56 MB TIF) [file ppat.1001053.s013.tif]

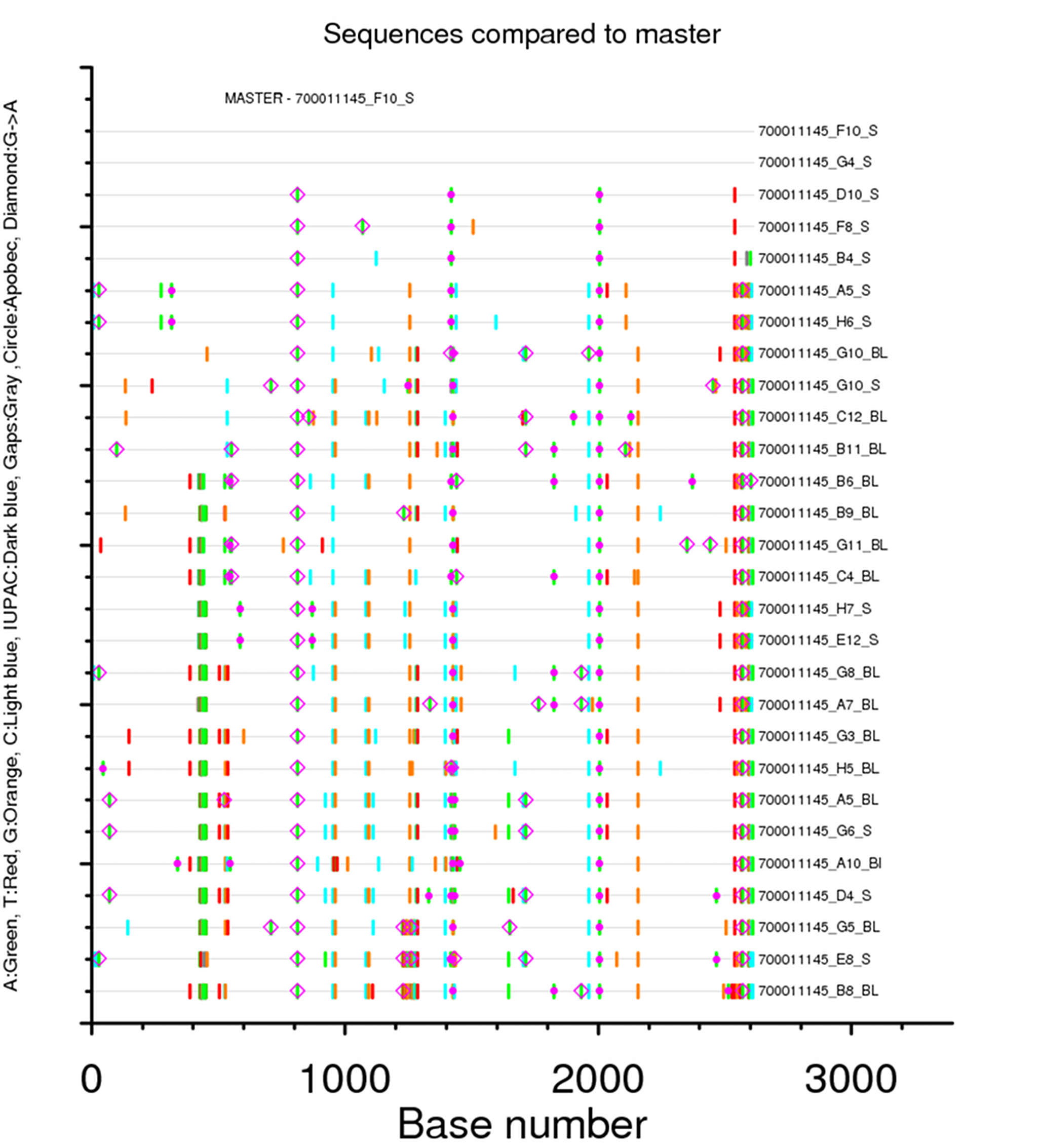

Supplement: Figure S14 — Highlighter plot for patient 700011145 with clonal amplification in the seminal tract. All blood (B) and semen (S) sequences are included. A representative amplified seminal variant is used as the master to illustrate identical sequences within the seminal plasma. Each vertical tic represents a mismatch from the master sequence as outlined in the figure. (2.27 MB TIF) [file ppat.1001053.s014.tif]

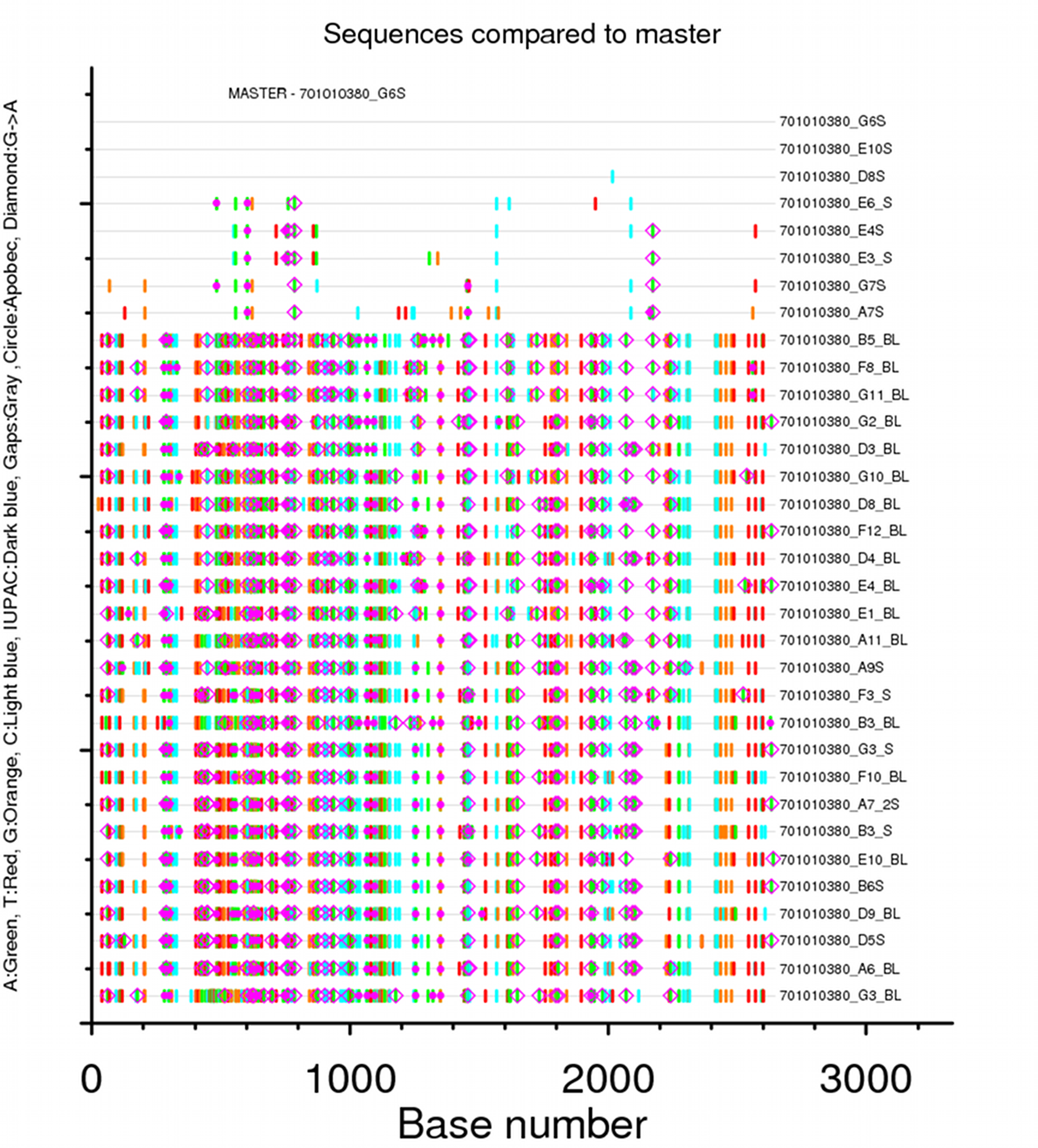

Supplement: Figure S15 — Highlighter plot for patient 701010380 with clonal amplification in the seminal tract. All blood (B) and semen (S) sequences are included. A representative amplified seminal variant is used as the master to illustrate identical sequences within the seminal plasma. Each vertical tic represents a mismatch from the master sequence as outlined in the figure. (4.05 MB TIF) [file ppat.1001053.s015.tif]
